# Targeting CRAF kinase in anti-cancer therapy: progress and opportunities

**DOI:** 10.1186/s12943-023-01903-x

**Published:** 2023-12-18

**Authors:** Penglei Wang, Kyle Laster, Xuechao Jia, Zigang Dong, Kangdong Liu

**Affiliations:** 1 Department of Pathophysiology, School of Basic Medical Sciences, Zhengzhou University, Zhengzhou, 450000 China; 2 Tianjian Laboratory for Advanced Biomedical Sciences, Zhengzhou, 450052 Henan China; 3 China-US (Henan) Hormel Cancer Institute, Zhengzhou, 450000 China; 4 Department of Pathophysiology, School of Basic Medical Sciences, China-US (Henan) Hormel Cancer Institute, AMS, College of Medicine, Zhengzhou University, 100 Kexue Avenue, Zhengzhou, 450001 Henan China; 5 Basic Medicine Sciences Research Center, Academy of Medical Sciences, Zhengzhou University, Zhengzhou, 450052 Henan China; 6 State Key Laboratory of Esophageal Cancer Prevention and Treatment, Zhengzhou University, Zhengzhou, 450000 Henan China; 7 Provincial Cooperative Innovation Center for Cancer Chemoprevention, Zhengzhou University, Zhengzhou, 450000 Henan China

**Keywords:** CRAF, RAF heterodimers, MAPK signaling pathway, Pan-RAF inhibitors, Combination therapy

## Abstract

The RAS/mitogen-activated protein kinase (MAPK) signaling cascade is commonly dysregulated in human malignancies by processes driven by *RAS* or *RAF* oncogenes. Among the members of the RAF kinase family, CRAF plays an important role in the RAS-MAPK signaling pathway, as well as in the progression of cancer. Recent research has provided evidence implicating the role of CRAF in the physiological regulation and the resistance to BRAF inhibitors through MAPK-dependent and MAPK-independent mechanisms. Nevertheless, the effectiveness of solely targeting CRAF kinase activity remains controversial. Moreover, the kinase-independent function of CRAF may be essential for lung cancers with *KRAS* mutations. It is imperative to develop strategies to enhance efficacy and minimize toxicity in tumors driven by *RAS* or *RAF* oncogenes. The review investigates CRAF alterations observed in cancers and unravels the distinct roles of CRAF in cancers propelled by diverse oncogenes. This review also seeks to summarize CRAF-interacting proteins and delineate CRAF's regulation across various cancer hallmarks. Additionally, we discuss recent advances in pan-RAF inhibitors and their combination with other therapeutic approaches to improve treatment outcomes and minimize adverse effects in patients with *RAF/RAS*-mutant tumors. By providing a comprehensive understanding of the multifaceted role of CRAF in cancers and highlighting the latest developments in RAF inhibitor therapies, we endeavor to identify synergistic targets and elucidate resistance pathways, setting the stage for more robust and safer combination strategies for cancer treatment.

## Introduction

The RAS-RAF-MEK signaling cascade plays a pivotal role in modulating cellular processes such as proliferation, differentiation, and survival. However, this pathway is often constitutively activated in human malignancies characterized by *RAS* or *RAF* oncogenic drivers. RAS proteins activate many signaling pathways through direct interaction with effectors and guanosine triphosphate-bound RAS (GTP-RAS). CRAF (RAF1), a member of the RAF kinase family, is an effector of RAS signaling that was first discovered in 1988. CRAF contributes to RAS signaling and exhibits an array of kinase-dependent and kinase-independent activities. A comprehensive understanding regarding the implication of aberrant CRAF activity in tumors remains unclear. However, a series of distinct characteristics among the RAF proteins potentially accounts for their varied roles in oncogenesis. In addition, it has been reported that the associations between ROK-α, ASK1, and MST2 with CRAF illuminate their joint contribution to the anti-apoptotic function of CRAF.

Focusing on the kinase-dependent and –independent role of CRAF could facilitate the discovery of new potential therapeutic strategies for cancer treatment. As such, developing chemotherapeutic CRAF inhibitors is an attractive area of research. Several CRAF/pan-RAF inhibitors with diverse structural and biochemical properties have recently entered preclinical and clinical development. As highlighted in previous research, endeavors to inhibit CRAF kinase activity in human malignancies have produced inconclusive outcomes. Furthermore, it is noteworthy that no selective CRAF inhibitors have received regulatory approval.

**Fig. 1:**
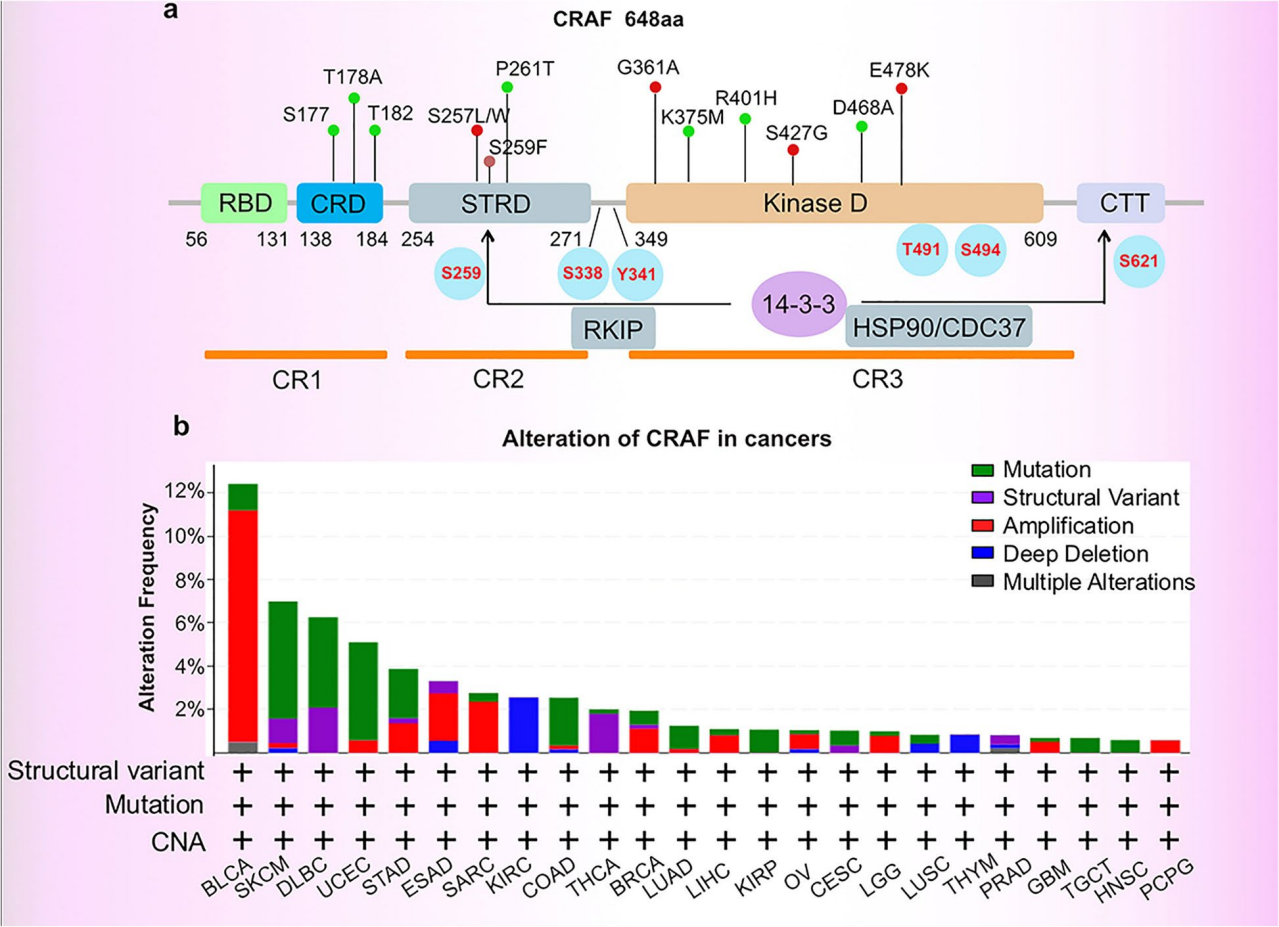
Structure and molecular alteration of CRAF in TCGA patient cohorts. **a** Three conserved regions (CR1–CR3) are indispensable in activating CRAF by RAS-GTP. CR1, located at the N-terminus of CRAF, is comprised of the Ras-binding domain (RBD) and cysteine-rich domain (CRD). The CRD maintains the auto-inhibited state of CRAF through interacting with 14-3-3 and the C-terminal kinase domain. The CR2 region consists of a serine-threonine-rich segment and recognizes a series of regulators, including 14-3-3, Hsp90, CDC37, and prohibitin. The auto-inhibited CRAF monomer requires a 14-3-3 dimer to bind to phosphorylated Ser 259 in the CR2 region. The CR3 region is comprised of the protein kinase domain and a short C-terminal tail harboring the second binding site for 14-3-3 proteins. Point mutations are depicted as small colored dots in the graph. Blue dots represent point mutations in CRAF that result in inhibitory effects, while red dots represent point mutations that lead to activating effects. **b** The alteration of CRAF based on TCGA Pan-cancer Atlas studies as visualized on the UniProt data platform. In the figure, the "+" symbols below each tumor type indicate that the bar graph analysis incorporates "structural variants", "mutations", and "Copy Number Alterations (CNA)" for that specific tumor type. BLCA, Bladder Urothelial Carcinoma; SKCM, Skin Cutaneous Melanoma; DLBC, Lymphoid Neoplasm Diffuse Large B-cell Lymphoma; UCEC, Uterine Corpus Endometrial Carcinoma; STAD, Stomach adenocarcinoma; ESAD, Esophageal adenocarcinoma; SARC, Sarcoma; KIRC, Kidney renal clear cell carcinoma; COAD, Colon adenocarcinoma; THCA, Thyroid carcinoma; BRCA, Breast invasive carcinoma; LUAD, Lung adenocarcinoma; LIHC, Liver hepatocellular carcinoma; KIRP, Kidney renal papillary cell carcinoma; OV, Ovarian serous cystadenocarcinoma; CESC, Cervical squamous cell carcinoma and endocervical adenocarcinoma; LGG, Lower Grade Glioma; LUSC, Lung squamous cell carcinoma; THYM, Thymoma; PRAD, Prostate adenocarcinoma; GBM, Glioblastoma multiforme; TGCT, Testicular Germ Cell Tumor; HNSC, Head and Neck squamous cell carcinoma; PCPG, Pheochromocytoma and Paraganglioma

**Fig. 2:**
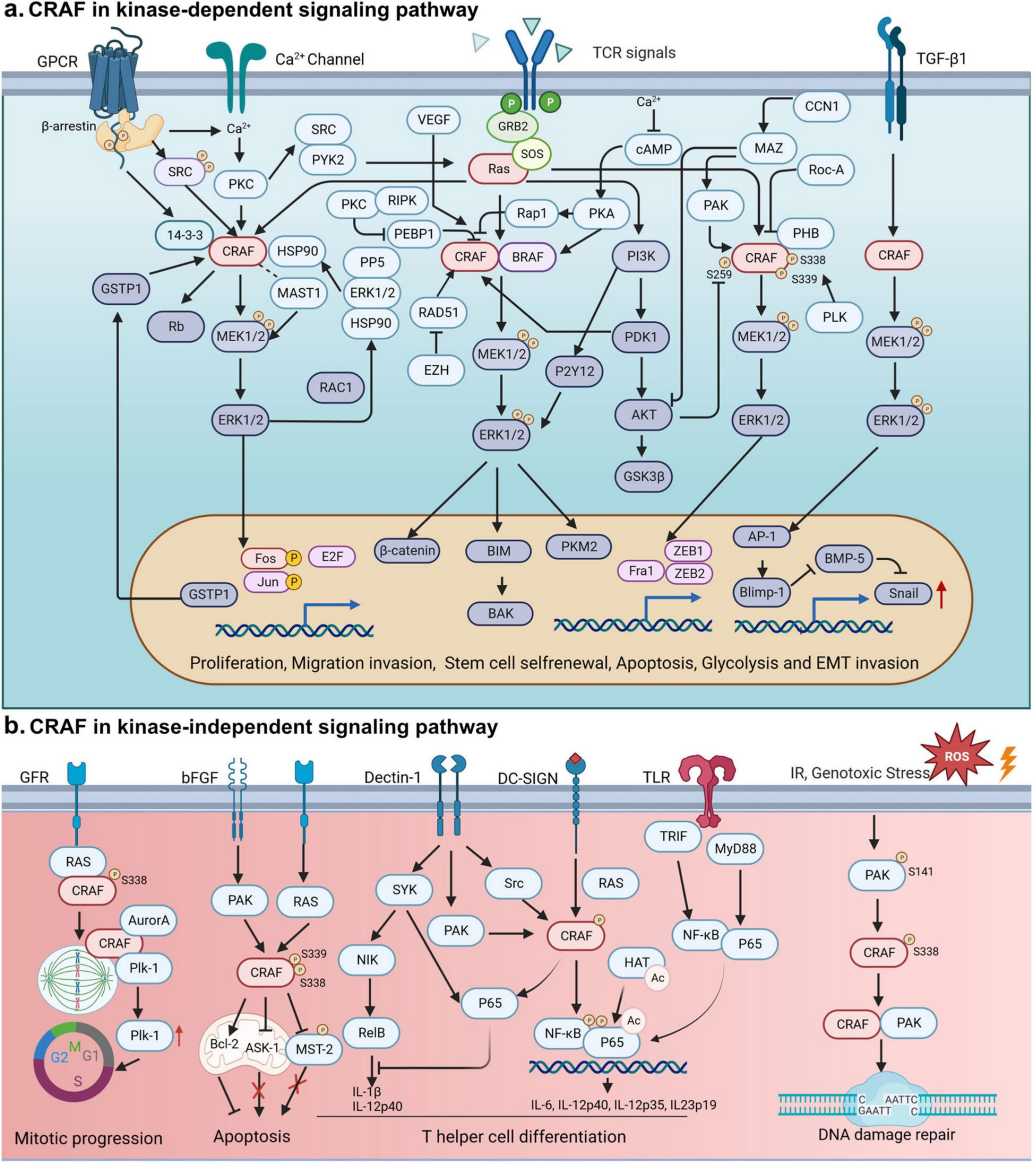
Kinase-Dependent and kinase-Independent Signaling Pathways Mediated by CRAF. **a** Role of CRAF in the kinase-dependent signaling pathway. As a cytosolic serine/threonine kinase, CRAF plays an important role in proliferation, migration invasion, EMT invasion, stem cell self-renewal, mitogen and stress-induced signaling responses, and cell apoptosis in the Ras-RAF-MEK-ERK cascade. β-arrestin mediates the active internalization of G protein-coupled receptors (GPCRs) and activates ERK1/2 through CRAF. GPCR also promotes Ca2^+^ mobilization and activation of protein kinase C (PKC) dependent of β-arrestin. Ca2^+^ signaling also promotes cAMP/protein kinase A (PKA) activity. PKA and PKC can activate B/CRAF, promoting the RAF/MEK/ERK MAPK signaling pathway. PKA can also facilitate ERK inhibition by forming an inactive complex with Rap1/CRAF. This complex disrupts the activation of MEK1 and MEK2 by sequestering CRAF activity. Similar to PKA, 14-3-3 proteins also contribute to the inactivation of CRAF. Upon activation of receptor tyrosine kinases (RTKs) by extracellular signals, CRAF dissociates from 14-3-3 and is recruited to the plasma membrane. PI3K-AKT is positioned downstream of RAS and interacts with CRAF through Polycystic Kidney Disease 1 (PKD1). The MAZ transcription factor is a downstream target of the oncoprotein Cyr61/CCN1 and promotes pancreatic cancer cell invasion via CRAF-ERK signaling. CRAF-MEK-ERK signaling pathway regulates numerous targets in the cytoplasm and nucleus, including c-FOS, c-JUN, E2F transcription factor, retinoblastoma protein (Rb), Bcl-2 interacting mediator of cell death (Bim) and Bcl-2 homologous killer (Bak), β-Catenin, Fos-related antigen 1(Fra1), ZEB1/ZEB2, and Pyruvate kinase M2 (PKM2). Glutathione S-transferase pi 1 (GSTP1) inhibits the CRAF pathway through an autocrine feedback loop. In addition, ERK can negatively regulate B/CRAF through the HSP90/ERK1/2/PP5 complex. Furthermore, Transforming Growth Factor-beta (TGF-β) regulates the AP-1-Snail involved in Epithelial-Mesenchymal Transition (EMT) through CRAF-MAPK signaling. **b** Role of CRAF in the kinase-independent signaling pathway. CRAF plays an important role in mitotic progression by promoting AURKA and Plk1 activation. Mitochondrial membrane-bound CRAF regulates cell apoptosis by recruiting Apoptosis Signal-Regulating Kinase 1 (ASK-1) and Bcl-2 phosphorylate homolog BAD. Moreover, mammalian sterile 20-like kinase (MST2)/Hippo signaling is also involved in anti-apoptotic. CRAF modifies T helper cell differentiation and enhances immune responses by antagonizing Spleen Tyrosine Kinase (Syk)-induced RelB activation. CRAF also induces acetylation of the Nuclear Factor-kappa B (NF-κB) p65 to modulate adaptive immunity by dendritic cells (DCs). Genotoxic stress also induces p21-activated protein kinase-1 (PAK-1) activity, activates CRAF at serine 338, and promotes DNA damage repair independent of MAPK pathway. GFR, Growth Factor Receptor; bFGF, basic Fibroblast Growth Factor; DC-SIGN, Dendritic Cell-Specific Intercellular adhesion molecule-3-Grabbing Non-integrin; TLR, Toll-like Receptor; TRIF, TIR-domain-containing adapter-inducing interferon-β; NIK, NF-κB-Inducing Kinase

**Fig. 3:**
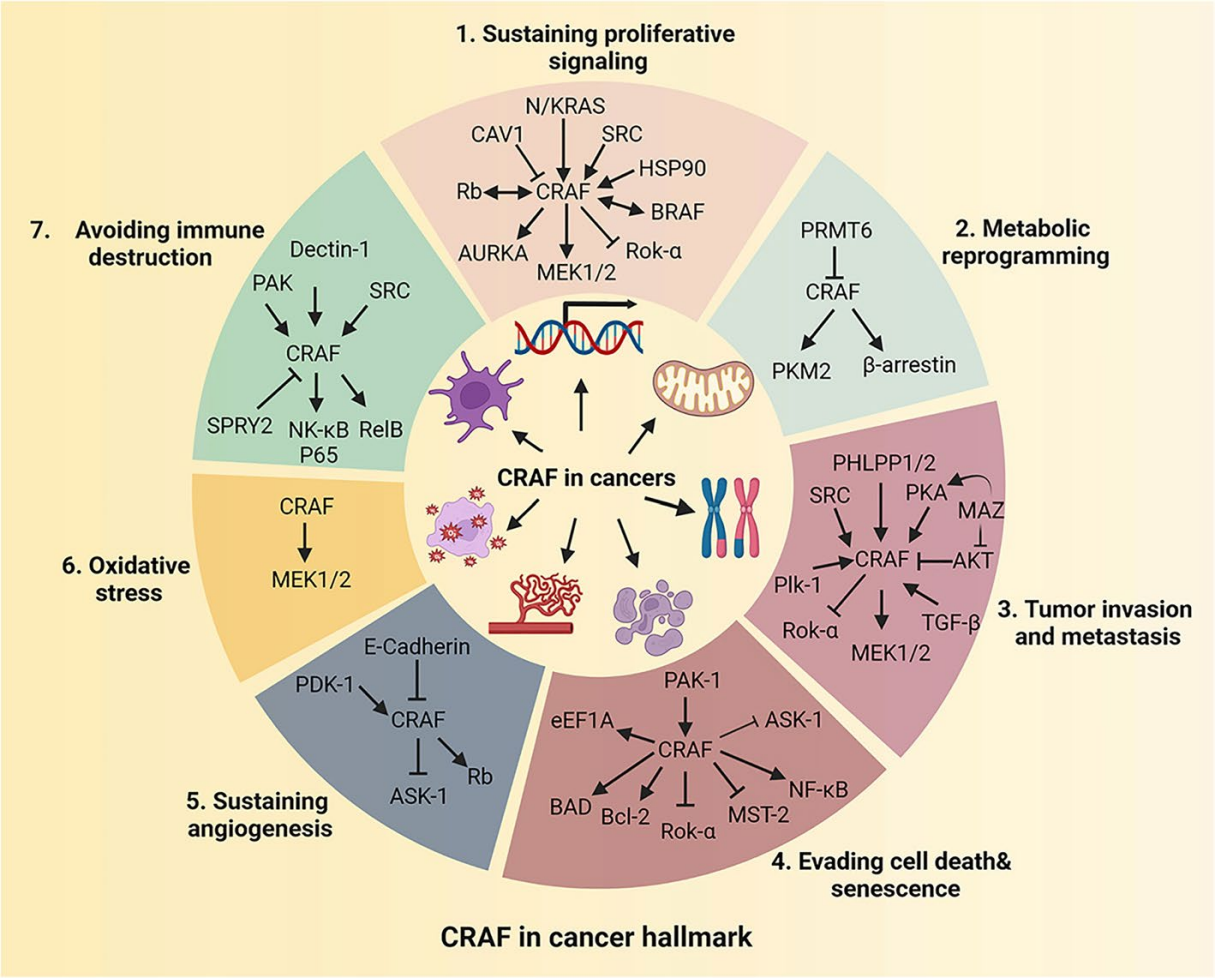
CRAF-mediated signal transduction promotes various cancer hallmarks. CRAF promotes seven features of malignant tumors, including self-sufficiency in growth signals, metabolic reprogramming (mainly glycolysis), tumor invasion and metastasis (EMT), evading cell death and senescence, sustaining angiogenesis, oxidative stress response, and avoiding immune destruction. The relevant upstream and downstream proteins are illustrated in the diagram. CAV1, Caveolin-1; HSP90, Heat Shock Protein 90; ROK-α, Rho-Associated Coiled-Coil Kinase Alpha; AURKA, Aurora Kinase A; PRMT6, Protein Arginine Methyltransferase 6; PKM2, Pyruvate Kinase M2; PHLPP1/2, PH Domain and Leucine-Rich Repeat Protein Phosphatases 1/2; PKA, Protein Kinase A; MAZ, MYC-Associated Zinc Finger Protein; PLK-1, Polo-Like Kinase 1; AKT, Protein Kinase B; TGF-β: Transforming Growth Factor-beta

**Fig. 4:**
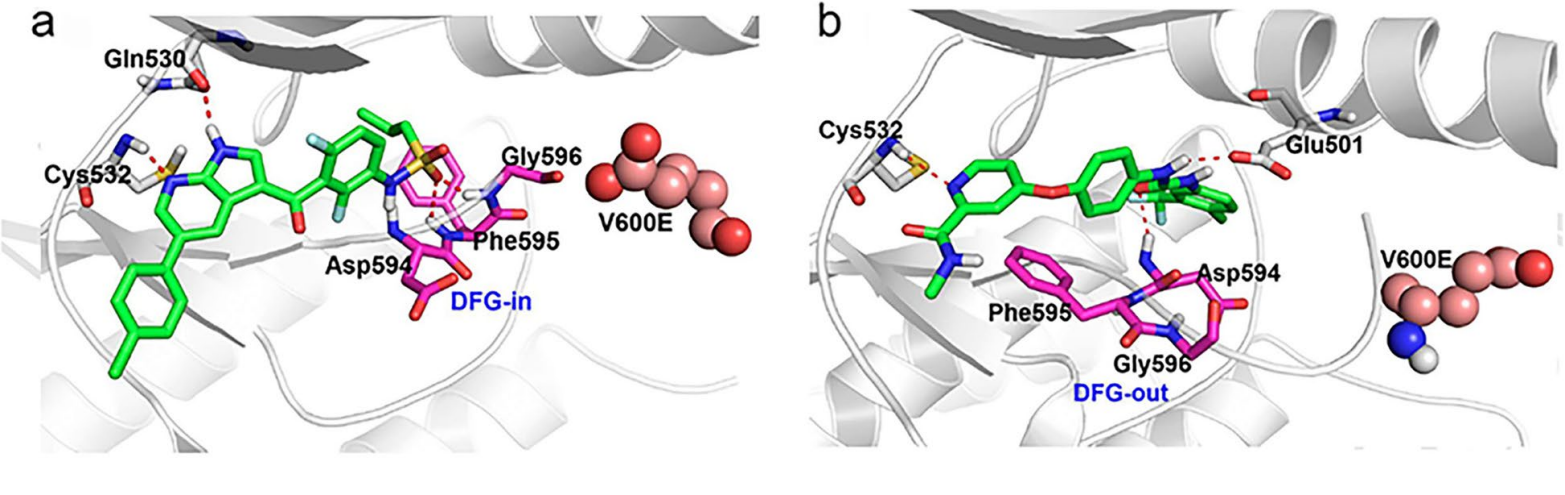
Binding mode of type I & II RAF inhibitors. **a** DFG-in conformation for PDB 3OG7 (crystalized with vemurafenib, specifically targets BRAF^V600E^ via selectively binding to the "active" DFG-in and αC-helix-out conformation of the ATP binding site); **b** DFG-out conformation for PDB 1UWJ (crystalized with sorafenib, "inactive" DFG-out and αC-helix-in conformation of the ATP binding site). This figure has been adapted from Wang, L. et al. [21]

**Fig. 5:**
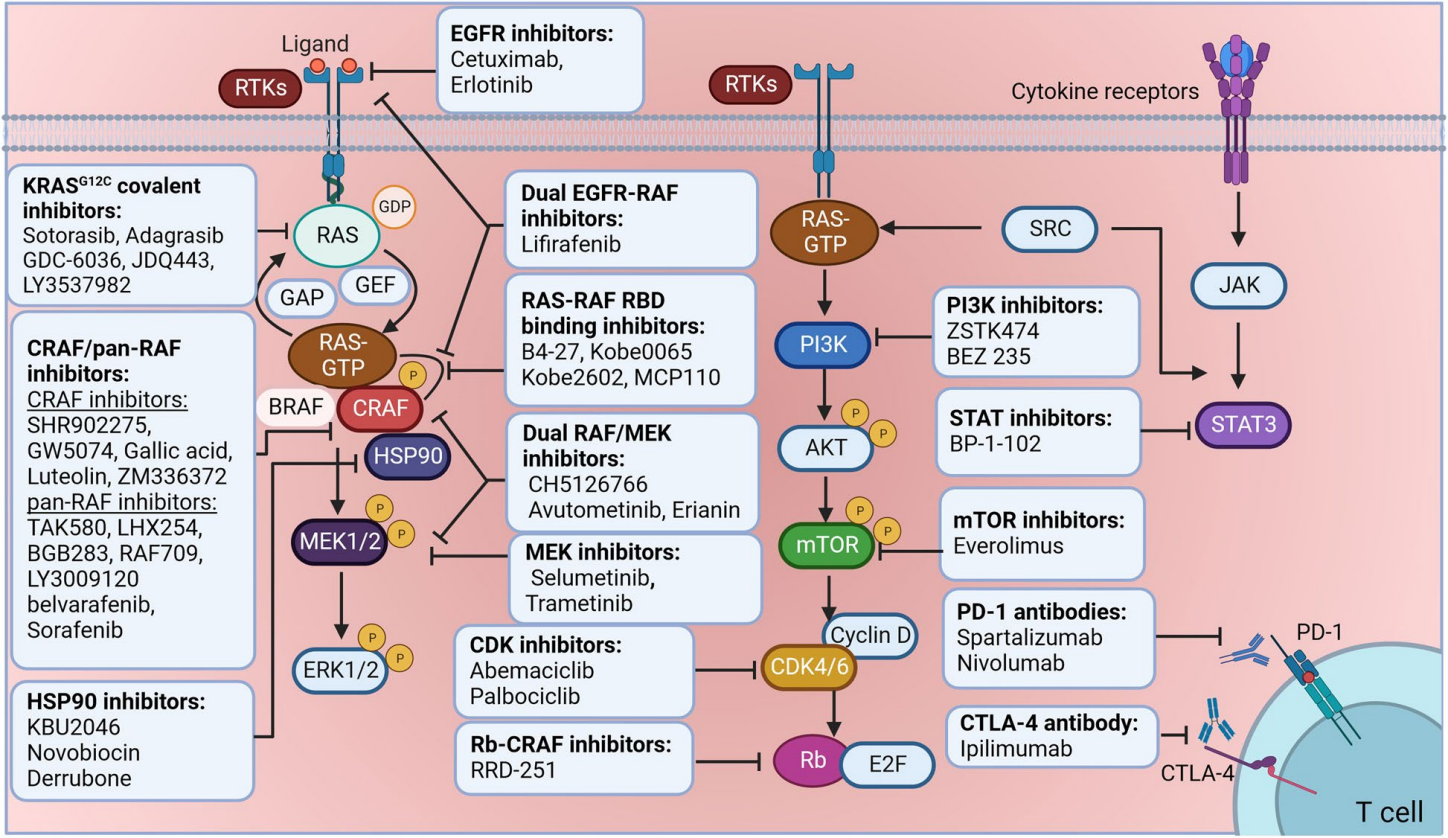
Combination therapies of CRAF/pan-RAF inhibitors and other treatments. Target therapies for CRAF/pan-RAF kinases (also refer to Table 3), including CRAF/pan-RAF inhibitors, Scaffold/chaperone proteins inhibitors, RAF RBD-RAS binding inhibitors, dual EGFR-RAF inhibitors, dual RAF-MEK inhibitors are illustrated above. Additionally, combination therapies of other treatments with CRAF/pan-RAF inhibitors, including RAS^G12C^ covalent inhibitors, EGFR inhibitors, MEK inhibitors, CDK inhibitors, Rb-CRAF inhibitors, PI3K inhibitors, STAT inhibitors, mTOR inhibitors, PD-1/PD-L1 antibodies, and CTLA-4 antibody are shown. EGFR, Epidermal Growth Factor Receptor; PI3K, Phosphoinositide 3-Kinase; STAT, Signal Transducer and Activator of Transcription; mTOR: Mammalian Target of Rapamycin; PD-1/PD-L1: Programmed Cell Death Protein 1/Programmed Death-Ligand 1; CTLA-4: Cytotoxic T-Lymphocyte-Associated Protein 4

This review describes documented *RAF1* alterations observed in several cancer types (Fig. 1, Tables 1 and 2). It further explores the contribution of CRAF's role in various kinase-dependent and kinase-independent signaling pathways (Fig. 2), and CRAF-interacting proteins in varied cancer hallmarks (Fig. 3). Our coverage of recent developments regarding pan-RAF inhibitors (Fig. 4), including the combination of RAF inhibitors with other types of inhibitors or treatment strategies, to enhance anti-cancer efficacy in diverse clinical settings is of particular significance (Fig. 5, Table 3). Overall, the present review aims to explore the role of CRAF in cancer and highlights recent advances in RAF inhibitor combination therapies to improve treatment efficacy and mitigate toxicities in patients with *RAF/RAS*-mutant tumors.

**Table 1:** Classification of selected *RAF1* point mutants

Mutations	Location	Kinase functional change	Ref.
D468A	Catalytic loop	Kinase dead	[1]
K375M	β3-K of K/E/D/D	Kinase dead	[1]
D486A	DFG-loop	Kinase dead	[2]
R391W	αC-helix	Kinase activated	[3]
P261A	Kinase domain	Oncogenic, sensitivity to combined type II RAF and MEK inhibitors	[4]
G361A	Glycine rich loop	Enhanced RAF dimerization and increased kinase activity, resistance to type I RAF inhibitors	[5]
S257W, S259F	CRD	Sensitivity to Sorafenib	[6]
S257P, P261T, G361A	CRD, Glycinerich Loop	Resistance to RAF inhibitors	[7]
E478K	Catalytic loop	Constitutively heterodimerize	[2]
E401H	Kinase domain	Defective in dimerization	[2]
S427G, I448V	Kinase domain	Activating variants	[8]

DFG-loop, Aspartate-Phenylalanine-Glycine loop; CRD, Carbohydrate Recognition Domain.

**Table 2:** Classification of oncogenic *RAF1* activating fusions

Fusion gene	Exon ratio (fusion gene/*RAF1*)	Associated cancers	Ref.
*MBNL-1*	EX 1-8 : EX 8-17	Langerhans cell histiocytosis	[9]
*TMF1*	EX 1-13 : EX 10-17	Sarcoma, NOS	
*QKI*	EX 1-3 : EX 8-17	Pilocytic Astrocytoma	
*SOX6*	EX 1-6 : EX 8-17	High-grade glioma, NOS	
*FYCO-1*	CRAF intron 5: FYCO-1 intron11	Multi-metastatic melanoma, sensitive to MEK inhibitors	[10]
*GOLGA-4*	EX 1-12 : EX 10-17; EX 1-21 : EX 8-17; EX 1-5/1-14 : EX 8-17; EX 1-17 : EX 8-17	Desmoplastic infantile ganglioglioma; Cutaneous melanoma, sensitive to MEK inhibitors; Melanoma; Pancreatic acinar cell carcinomas	[9, 11–13]
*NFIA*	EX 1-6 : EX 9-17	Pilocytic astrocytoma	[14]
*SRGAP3*	EX 1-10 : EX 9-17; EX 1-12 : EX 10-17	Low grade glioma, NOS; Pilocytic astrocytoma	[9, 15]
*LRCH3*	EX 1-12/1-13 : EX 8-17	Melanoma	[12]
*CTDSPL*	EX 1-2 : EX 8-17	Melanoma	
*MAP4*	EX 1-13/1-15 : EX 8-17	Melanoma	
*PRXAR2A*	EX 1-8/1-9 : EX 8-17	Melanoma	
*CTNNA1*	EX 1-6 : EX 8-17	Pancreatic acinar cell carcinomas	[11]
*GATM*	EX 1-2 : EX 10-17	Pancreatic acinar cell carcinomas	
*PDZRN3*	EX 1-5 : EX 8-17	Pancreatic acinar cell carcinomas	
*HERPUD1*	EX 1-8 : EX 8-17	Pancreatic acinar cell carcinomas	
*TRIM33*	EX 1-11 : EX 8-17	Pancreatic acinar cell carcinomas	
*LRRFIP2*	EX 1-20 : EX 8-17	Acral Melanoma	[16]
*PDZRN3*	EX 1-5 : EX 10-17	Spindle cell tumors	[17, 18]
*SLMAP*	EX 1-10 : EX 8-17	Spindle cell tumors	[17]
*MTAP*	EX 1-7 : EX 8-17	Soft tissue sarcoma	[19]
*ATG7*	EX 1-18 : EX 8-17	Glioblastoma; Anaplastic pleomorphic xanthoastrocytoma	[9, 20]

*MBNL-1* Muscleblind-like 1, *TMF1* TATA Element Modulatory Factor 1, *QKI* Quaking, *SOX6* SRY-Box Transcription Factor 6, *FYCO-1* FYVE and Coiled-Coil Domain Autophagy Adaptor 1, *GOLGA-4* Golgin A4, *NFIA* Nuclear Factor I/A, *SRGAP3* SLIT-ROBO Rho GTPase Activating Protein 3, *LRCH3* Leucine-rich Repeat-containing Protein 3, *CTDSPL* CTD Small Phosphatase-Like Protein, *MAP4* Microtubule-Associated Protein 4, *PRXAR2A* Peroxiredoxin-1 Antioxidant Response Element 2A, *CTNNA1* Catenin Alpha-1, *GATM* Glycine Amidinotransferase, *PDZRN3* PDZ Domain-Containing Ring Finger 3, *HERPUD1* Homocysteine-Inducible ER Protein with Ubiquitin-Like Domain 1, *TRIM33* Tripartite Motif-Containing 33, *LRRFIP2* Leucine-Rich Repeat Flightless-Interacting Protein 2, *SLMAP* Sarcolemmal Membrane-Associated Protein, *MTAP* Methylthioadenosine Phosphorylase, *ATG7* Autophagy-Related 7, *NOS* Not Otherwise Specified

**Table 3:** Target therapies for CRAF/pan-RAF kinases

Compounds	Targets (IC_50_ or Kd values)	Administration efficiency		Clinical trial status and efficacy	Ref.
		In vitro	In vivo usage		
**Selective CRAF inhibitors**					
GW 5074	9 nM - CRAF	GW5074 potentiates the cytotoxicity of Sorafenib through mitochondrial dysfunction	GW5074 (25 mg/kg, IP) combination with Sorafenib in ACHN RCC tumors	NCT03406364, Phase I , Combined GW5074 and Sorafenib to treat solid tumor	[22–24]
ZM 336372	0.07 μM - CRAF	ZM336372 suppresses carcinoid tumor cell proliferation and induces cell cycle inhibitors p21 and p18	NO	NO	[25]
SHR902275	1.6 nM - CRAF 5.7 nM - BRAF V600E 10 nM - BRAF WT	SHR902275 shows cell growth inhibition with GI_50_ of 1.5 and 0.17 nM, 0.4 nM, and 0.32 nM for H358, A375, Calu6, and SK-MEL2 cells	SHR902275 (3-30 mg/kg, orally) inhibits cancer progression in *RAS* mutant Calu6 CDX model	NO	[26]
RAF inhibitor 2t	50 nM - CRAF	2t exhibited potent activities on WM3629 cell lines (IC_50_ 0.56–0.86 μM)	NO	NO	[27]
RAF inhibitor 10c	8.79 nM - CRAF 38.3 nM - BRAF V600E	10c were (IC_50_ 1.82 μM and 2.73 nM) against the A375P and U937 cell lines* in vitro*	NO	NO	[28]
RAF inhibitor 7a	-	7a exhibited activities on A375P and WM3629 (IC_50_ 0.62 μM and 4.49 μM)	NO	NO	[29]
RAF inhibitor 10d	38.6 nM - CRAF 9.45 μM - BRAF WT	10d exhibited activities on A375P and WM3629 (IC_50_ 15.93 μM and 0.65 μM)	NO	NO	[30]
**RAF RBD-RAS binding inhibitors**					
Kobe0065	46 ± 13 μM - KRAS G12V	Kobe0065 exhibits inhibitory activity toward HRas-CRAF binding	Kobe0065 (80-160 mg/kg, orally) inhibits activity on SW480 CDX harboring the KRAS G12V mutation	NO	[31]
Kobe2602	149 μM - KRAS G12V	Kobe2602 exhibits inhibitory activity toward HRas-CRAF binding	Kobe2602 (80 mg/kg, orally) exhibits antitumor activity on SW480 CDX harboring the KRAS G12V mutation	NO	[31]
MCP110	-	MCP110 (20 μM) significantly inhibits Ras-mediated stimulation of CRAF activity in fibrosarcoma HT1080 cells	NO	NO	[32]
**CRAF Scaffold/chaperone protein inhibitors**					
KBU2046	HSP90	KBU2046 (10 μmol/L) inhibits intracellular activation of CRAF, thereby achieving selective inhibition of cell motility.	KBU2046 (150 mg/kg, orally) with ZA (100 μg/kg, IP) targeting strategy	NO	[33]
Novobiocin	HSP90	Novobiocin (0.8 mM) displayed a reduced cellular CRAF activity but not BRAF V600E	NO	Alterations of DNA Repair genes in solid neoplasm, NCT05687110, Phase 1, Recruiting	[34]
17-DMAG	62 ± 29 nM - HSP90	17-DMAG (1 μM) reduces the kinase activity of CRAF and BRAF V600E	17-DMAG (10 or 20 mg/kg, IP) in Prostatic cancer	NCT00803556, Phase 1, Completed; NCT00089362, Phase 1, Completed; NCT00248521, Phase 1 Active, not recruiting	[34]
Locostatin	RKIP	Locostatin (200 µM) binds RKIP protein and disrupts the interaction between RKIP, CRAF, and GRK2	NO	NO	[35]
**Pan-RAF dimers selective inhibitors**					
RAF709	0.5 nM - CRAF 0.4 nM - BRAF	RAF709 stabilizes BRAF-CRAF dimers (EC_50_ 0.8 μM), inhibition proliferation of Calu-6 cells (EC_50_ 0.95 μM)	RAF709 (30-200 mg/kg, orally) results in Calu-6 tumor regression	NO	[36]
TAK-632	1.4 nM - CRAF, 2.4 nM - BRAF V600E 8.3 nM- BRAF WT	TAK-632 shows antiproliferative effects both in A375 (GI_50_ of 40-190 nM) and SK-MEL-2 (GI_50_ of 190-250 nM) cells	TAK-632 (60-120 mg/kg, orally) exhibits an antitumor effect without toxicity in SK-MEL-2 melanoma	NO	[37]
LXH254	0.072 nM - CRAF 0.21 nM - BRAF 6.4 nM - ARAF	LXH254 (0-10 µM) inhibits both monomeric and dimeric RAF and promotes RAF dimer formation; More sensitivity to ARAF depletion cells	LXH254 (100 mg/kg, orally) decreased tumor-harboring BRAF mutations with or without activated NRAS or KRAS	NCT04294160, Phase 1, BRAF V600 Colorectal Cancer, Active, not recruiting; NCT02607813, Phase 1, NSCLC/Ovarian Cancer/Melanoma/Solid Tumors, Terminated; NCT02974725, Phase 1, NSCLC, Active, not recruiting	[38, 39]
LY3009120	15 nM - CRAF 5.8 nM - BRAF V600E 9.1 nM- BRAF WT	LY3009120 exhibits anti-proliferative effects on cell lines harboring BRAFV600E, KRAS^G13^ and KRAS^G12^ mutations	LY3009120 (20 mg/kg, orally) inhibits BRAF and KRAS mutant CRC CDX; (15 or 30 mg/kg, orally) in the H2405 model	NCT02014116, Phase 1, Advanced or Metastatic Cancer, Terminated	[40, 41]
Belvarafenib	5 nM - CRAF 56 nM - BRAF WT 7 nM - BRAF V600E	Belvarafenib effect in BRAF- and NRAS-mutant tumors, but acquired ARAF mutations drive resistance	Belvarafenib reduced tumor burden in mice with A375SM melanoma.	NCT04835805, Phase 1, NRAS mutant Advanced Melanoma; NCT03118817, Phase 1, Solid Tumor; NCT04589845, Phase II, Solid Tumors; NCT02405065, Phase 1, Neoplasms	[42]
**CRAF Mult-kinase inhibitors**					
Sorafenib	6 nM - CRAF 20 nM - BRAF 15 nM - VEGFR3 20 nM - PDGFRβ 57 nM - FLT3 58 nM - c-Kit	Sorafenib (0.01 to 3 μM) blocks MAPK pathway with MEK 1/2 and ERK 1/2 phosphorylation (IC_50_, 40 and 100 nM, respectively)	Sorafenib (30-60 mg/kg, orally) produces broad spectrum antitumor activity in colon, breast, and non-small-cell lung cancer xenograft models	NCT04387695, Phase 3, Unresectable Hepatocellular Carcinoma|Portal Vein Thrombosis; NCT03456401, Phase 2, Renal Cancer; NCT01715441, Phase 2, Metastatic Colorectal Cancer With KRAS Mutation	[22, 24, 43]
RAF265	RAF/VEGFR2	RAF265 inhibit cell viability of HT29 and MDAMB231 cells (IC_50_ values of 5 to 10 μM)	RAF265 (30 mg/kg qd, single use) and combination with RAD001 (both 12 mg/kg qd) in HCT116 xenografts	NCT00304525, Phase 1/2, Metastatic Melanoma;NCT01352273, Phase 1, Advanced Solid Tumors	[44, 45]
Avutometinib	8.2 nM - BRAF V600E 56 nM - CRAF 160 nM - MEK 190 nM - BRAF	Avutometinib inhibits activation of ERK2 by MEK1 (IC_50_ of 160 nM) and activation of MEK1 by CRAF (IC_50_ of 56 nM)	Single or in combination with PD0325901 in HCT116 (KRAS-mutant) models, the ED50 for Avutometinib and PD0325901 are 0.056 and 0.80 mg/kg, respectively	NCT05669482 (Phase 1/2), KRAS Activating Mutation, Metastatic Cancer, Pancreas Cancer, Neoplasms Pancreatic Malignant Neoplasm of Pancreas	[46]
Regorafenib	2.5 nM - CRAF 13/4.2/46 nM - VEGFR1/2/3 22 nM - PDGFRβ 7 nM - Kit 1.5 nM - RET	Regorafenib (0-10 μM) exhibits anti-proliferation activity in GIST 882, Thyroid TT, MDA-MB-231, HepG2, A375, and SW620 cells; as well as in Hep3B ( IC50 of 5 μM)	Regorafenib (10 mg/kg, Orally) inhibits rat GS9L glioblastoma model; (0-100 mg/kg, Orally) exhibits antitumorigenic and antiangiogenic effects in the Colo-205, MDA-MB-231, and 786-O model	NCT03465722, Phase 3, GIST; NCT01774344, Phase 3, Carcinoma, Hepatocellular; NCT02788279, Phase 3, Colorectal Cancer; NCT01271712, Phase 3, Gastrointestinal Stromal Tumors; NCT01103323, Phase 3, Metastatic Colorectal Cancer	[47–49]
Erianin	CRAF/MEK	Erianin exhibits anti-proliferation effect in A375 (12.0 ± 0.9 nM), SK-MEL-28 (50.6 ± 1.7 nM), SK-MEL-2 (59.7 ± 7.2 nM) and HCT116 (20.6 ± 2.2 nM)	Erianin (50 mg/kg, Orally) inhibits A375, SK-MEL-28, SK-MEL-2 and HCT116 xenografts and melanoma/CRC patient derived tumor xenografts	NO	[50]

*ACHN* Adenocarcinoma of the Kidney, *RCC* Renal Cell Carcinoma, *CDX* Cell-Derived Xenograft, *IP* intraperitoneal injection, *NSCLC* Non-Small Cell Lung Cancer, *PDAC* Pancreatic Ductal Adenocarcinoma, *CRC* Colorectal Cancer, *HSP90* Heat Shock Protein 90, *RKIP* Raf Kinase Inhibitor Protein, *GRK2* G Protein-Coupled Receptor Kinase 2, *VEGFR3* Vascular Endothelial Growth Factor Receptor 3, *PDGFRβ* Platelet-Derived Growth Factor Receptor Beta, *FLT3* FMS-Like Tyrosine Kinase 3, *c-Kit* Stem cell factor receptor, *RET* Rearranged During Transfection, *q.d.* Quaque die (Latin)

## Structures of CRAF proteins

The RAF family, consisting of three RAF kinase paralogs: A-, B-, and CRAF, function as downstream effectors of RAS. Of the three RAF isoforms, CRAF is the earliest discovered RAF paralog. Three conserved regions (CR1–CR3) are indispensable for the recruitment and activation of CRAF by upstream effectors. Specifically, the CR1 region, comprised of the Ras-binding domain (RBD) and the cysteine-rich domain (CRD), is mainly responsible for binding to the RAS and membrane phospholipids. RBD and CRD of CRAF are associated with membrane-bound RAS via multivalent and dynamic interactions [51]. Cytosolic monomer RAF is auto-inhibited through the spatial conformation of the N-terminal regulatory region to the C-terminal kinase domain [52] and is activated by the recruitment of RAS-GTP to the plasma membrane. It is widely recognized that the RBD and CRD are two distinct globular domains that play crucial roles in the activation of CRAF. RBD binds to the interface of the RAS G domain, while CRD is responsible for the association with anionic lipid-rich membranes. Recent evidence has revealed synergistic influences of RBD and CRD on the dynamics of cellular membranes. The recruitment of RBD in proximity to the plasma membrane augments the local concentration of anionic lipids, thereby potentially intensifying the surface interaction between the RBD-CRD construct and the membrane [53]. Besides anchoring CRAF to the plasma membrane, CRD binds to RAS and stabilizes the active RAS-RAF complex in an RBD-independent manner [54]. A previously published report illustrated that CRD maintains the auto-inhibited state of CRAF through interacting with 14-3-3 and the C-terminal kinase domain [55]. CRD also plays a crucial role in RAF activation independently of its role in binding to RAS. Timothy et al. [56] revealed that the *RAF1* T178A mutation located in the CRD domain diminished the interaction with RAS and inhibited CRAF kinase activity (~50%). Similarly, Daub et al. found that the CRD p.S177 and p.T182 mutation also resulted in impaired kinase activation [57].

The CR2 region, composed of a serine-threonine-rich segment, is recognized by various regulators, including 14-3-3, HSP90, CDC37, and prohibitin [58–60]. Auto-inhibited monomeric CRAF requires a 14-3-3 dimer binding to phosphorylated Ser259 in the CR2 region. Dephosphorylation of the CRAF Ser259 residue by HSP90, prohibitin, or protein phosphatase 2A (PP2A) abolishes the inhibitory effect of 14-3-3, resulting in its dissociation from the scaffold protein and its subsequent transfer to the plasma membrane for activation. CR3 contains the protein kinase domain and the remaining C-terminal tail, which harbors the second binding site that anchors the 14-3-3 scaffold protein. The catalytic kinase domain contains an αC-helix in the N-lobe, catalytic loop, and activation segment (AS) in the C-terminal, which spatially regulates CRAF kinase activity [61]. A recent study indicated that aside from classical catalytic activity, the CRAF kinase domain can also interact with the plasma membrane, thus coordinating CRAF recruitment and modulating its activation [62]. In summary, a detailed understanding of these conserved regions of CRAF is crucial for the development of more effective and safer CRAF inhibitors for cancer treatment. Key areas of focus include the synergistic effects of the RAS-binding domain and the cysteine-rich domain, the role of the CR2 region in binding to 14-3-3 proteins for activation, and the potential of the kinase domain to interact with the membrane.

## CRAF functions and related pathways in oncogenic-driven cancers

Immature CRAF polypeptides are translated from the ribosome, followed by proper folding and stabilization by complex chaperones HSP90 and CDC37 [59]. Cytosol-localized monomeric RAF is auto-inhibited through the physical association of the N-terminal regulatory region to the C-terminal kinase domain. Moreover, 14-3-3 dimers contribute to steady-state regulation by binding to CRAF Ser259 and Ser621 residues located at the CR1 and CR3 regions, respectively. Upon activation of membrane-bound receptor tyrosine kinases (RTKs) by extracellular stimuli, CRAF becomes dissociated from 14-3-3 and is recruited to the plasma membrane to facilitate the propagation of downstream signaling [63]. The SHOC2–MRAS–PP1C complex facilitates the dissociation of 14-3-3 from CRAF through the dephosphorylation of Ser259 within the N-terminal domain. SHOC2-mediated dephosphorylation of CRAF is essential for RAF dimerization and efficient activation of the ERK pathway [64]. Additionally, the scaffold protein prohibitin facilitates the displacement of 14-3-3 from Ser259, further facilitating CRAF activation [65–67]. Several phosphorylation sites within or flanking the CRAF kinase domain are involved in its activation (Fig. 1). Thr491 and Ser494 sites within the activation segment are phosphorylated following CRAF membrane localization [68]. Ser338 and Tyr341 are considered the most essential phosphorylation sites for fully activating CRAF [69]. However, Oehrl W. et al. demonstrated that phosphorylation at Ser338 is not essential for CRAF activation, suggesting that CRAF activation can occur in a kinase-independent manner [70]. Taken together, CRAF acts as a key effector in the canonical RAS-MAPK cascade and plays a central role in kinase-independent signaling pathways in different cancers (as depicted in Fig. 2a and b).

### The role of CRAF in cancers with mutant *RAS*

*RAS* mutations are the most common alterations in MAPK signaling and occur in nearly 30% of all human cancers. According to statistics, *KRAS* mutations exist in more than 90% of pancreatic ductal adenocarcinomas (PDACs), 40% of colorectal cancers, and 35% of non-small cell lung cancers (NSCLCs) [71]. Moreover, *NRAS* mutations occur in approximately 20% of malignant melanomas. Although RAS has historically been described as an "undruggable" target, allele-specific KRAS^G12C^ inhibitors have shown clinical benefits in lung cancer patients [72]. Additionally, non-covalent pan-KRAS inhibitors display promising therapeutic potential for patients with *KRAS*-driven malignancies [73].

Interestingly, a growing body of evidence coincides with the notion that the RAF family, particularly CRAF, assumes a pivotal role in oncogenic *KRAS*-driven cancers. An examination of CERES scores among *KRAS, NRAS*, and *BRAF*^V600E^ mutant cancer cell lines indicated that *KRAS* and *NRAS* mutant cells had a heightened reliance on CRAF for proliferation, while *BRAF*^V600E^ mutant cells primarily depended on BRAF for their growth [74]. Furthermore, genetic analysis of the RAS effectors within the MAPK pathway has revealed that ablation of CRAF exerts a promising therapeutic response with acceptable toxicities [75, 76]. Nonetheless, the impact of CRAF on tumorigenesis differs markedly across various *KRAS*-driven tumor models, and the exact role of CRAF in *KRAS*-mutant tumors remains to be elucidated.

#### *KRAS*-mutant lung cancer

Several studies have demonstrated that CRAF plays a crucial role in the development of lung carcinoma driven by the *KRAS* oncogene. Karreth et al. confirmed that CRAF, not BRAF, was essential for tumor initiation by resident *KRAS*^G12D^ oncogenes in non-small cell lung carcinoma. Interestingly, while BRAF has been proposed as the primary ERK activator due to its higher kinase activity [77], knock-out of BRAF and/or CRAF did not impact the phosphorylation of MEK [74, 78, 79]. Furthermore, systemic depletion of both CRAF and BRAF kinases in adult mice was found to be well-tolerated [80]. This suggests that in *KRAS*^G12V^ driven NSCLCs, all RAF proteins (A/B/CRAF) can sustain mitogenic signaling through the MAPK pathway.

Furthermore, recent research has shown that the *KRAS* mutant lung cancer growth is driven by heterodimerization of CRAF and ARAF, not merely by CRAF kinase activity. Remarkably, depletion of CRAF and ARAF inhibits sustained MAPK activation and alleviates cell-cycle arrest caused by CRAF ablation [74]. Moreover, CRAF ablation was shown to limit reactions detrimental to maintaining homeostasis [79, 80]. Concomitant suppression of CDK4 kinase activity and CRAF ablation effectively induced complete regression in 25% of *KRAS/TP53*-driven lung cancers [81]. Either CRAF depletion or sorafenib treatment decreased cyclin E expression and induced G1 arrest in *KRAS* mutant NSCLC cells [43]. Impairment of CRAF-MEK complex formation enhanced inhibition of CRAF-dependent ERK signaling in *KRAS* mutant tumors [82]. One promising hypothesis suggests that therapeutic effects derived from CRAF ablation may rely on other mechanisms aside from kinase inhibition. Although the contribution of RAF isoforms to the various stages of *RAS*-driven tumorigenesis and development remains unclear, the depletion of CRAF from *KRAS*^G12V^ expressing lung cells completely inhibited tumor development without inducing significant toxicities, suggesting a potential role for CRAF in modulating an alternative pathway essential for malignant transformation [43, 80]. Sanclemente et al. demonstrated that the anti-proliferative effect observed upon CRAF attenuation in lung adenocarcinoma cells occurs through occluding its interaction with the ASK1 or MST2 kinases [1]. Moreover, the enhanced apoptotic activity stemming from the loss of CRAF expression has a minimal impact on normal tissue homeostasis [83]. These studies suggest that targeting CRAF might be a beneficial therapeutic approach for *KRAS* mutant lung cancers. Moreover, dimerization of CRAF, rather than its kinase activity, is essential for *KRAS* mutant-driven lung cancer [83]. Depleting CRAF inhibited tumor growth in *KRAS/p53*-driven lung tumors. However, expressing kinase-dead CRAF variants (CRAF^D468A^ and CRAF^K375M^) in *KRAS/p53*-driven lung GEMM models failed to achieve the same effect [1].

Above all, in *RAS*-driven lung cancer, inhibition of CRAF kinase activity with selective inhibitors remains suboptimal due to its less prominent role in the RAS-MAPK signaling pathway and paradoxical activation of other RAF subtypes. Regardless, the intervention of RAF dimers and promoting CRAF degradation may be an effective therapeutic strategy for *KRAS* mutant lung cancers.

#### *KRAS*-mutant pancreatic carcinomas

While some reports suggest that disrupting the PHB-CRAF interaction could impair oncogenic *RAS*-driven pancreatic cancer through the ERK-MAPK signaling pathway [67], it is widely believed that merely deleting CRAF produces minimal effects in *KRAS*-mutated pancreatic ductal adenocarcinomas (PDAC). Eser et al. found that CRAF expression was not essential for the initiation of *KRAS*-driven PDAC [84]. The function of CRAF in PDAC markedly differs from its role in *KRAS* mutant lung cancer, and the underlying mechanism for the disparity remains elusive. Cell proliferation defects in *KRAS* mutant pancreatic cancer cells in response to CRAF inhibition occur without p-ERK attenuation [74] and may be attributed to the differences in the kinase-dependent and kinase-independent roles of CRAF in *KRAS* mutant lung cancer compared to *KRAS* mutant PDAC. However, ablation of EGFR/CRAF resulted in complete regression of PDAC with mutant *KRAS/TP53* [85]. Moreover, the adverse effects from concurrently depleting EGFR and CRAF mirrored those observed in EGFR-deficient mice, suggesting such an approach may be well-tolerated in vivo [86]. In a parallel study, Assi et al. also found that CRAF/EGFR signaling is crucial for pancreatic tumorigenesis in adult pancreas harboring *KRAS* mutations [87]. In addition, although pan-RAF inhibitors elicit unacceptable toxicities in the clinic when combined with MEK inhibitors by affecting the MAPK pathway [40], researchers have suggested that a low-dose intervention of pan-RAF and ERK inhibitors could provide an effective therapeutic alternative for *KRAS* mutant PDAC by circumventing harmful feedback mechanisms associated with ERK reactivation [88].

#### *KRAS*-mutant colorectal carcinomas

Solely inhibiting CRAF is insufficient to suppress the MAPK signaling and the proliferation of colon cancer cells harboring the *KRAS*^G13D^ mutation [89]. This suggests that the removal of CRAF has minimal impact on MAPK signaling, which is likely maintained by BRAF. However, Borovski et al. posited that CRAF is crucial for sustaining the transformed phenotype of *KRAS* mutant CRC cells, exerting its effects in a kinase-dependent manner but independently of MEK [90]. While *KRAS* mutations are notably frequent in lung adenocarcinomas (14%) and colorectal tumors (5%) [71], their dependence on the *KRAS* mutation seems to differ between these cancers. Specifically, a series of phase I/II clinical trials using AMG 510 or MRTX849, both KRAS^G12C^ inhibitors, produced significant responses in approximately half of the lung cancer patients, yet yielded no comparable results for those with colon tumors [91, 92]. Depletion of CRAF induces apoptosis in colon cancer cells by activating *RAS* mutations via a MEK-independent RAF signaling pathway. When combined with simultaneous MEK kinase inhibition, the pro-apoptotic effect is amplified [93]. The role of CRAF in mediating tumor growth in *KRAS* mutant lung cancer, pancreatic cancer, and colon cancers is gradually gaining consensus in the scientific community [74]. It is clear that the kinase domain of CRAF, independent of its catalytic activity, plays a significant role in this process. Interestingly, the mechanism that necessitates CRAF heterodimerization with ARAF is crucial for maintaining *KRAS*-driven tumors [74]. Consequently, the kinase-independent role of CRAF is pivotal when considering combination therapies targeting *KRAS*-driven colon cancer. The precise mechanisms behind the kinase-dependent and -independent activities of CRAF in *RAS*-driven cancers remain to be fully elucidated.

#### *RAS*-mutant skin cancer

CRAF was reported to play a vital cell-autonomous role in the development and maintenance of *RAS*-driven skin tumors. CRAF was reported as the primary RAS effector signaling through ERK specifically in melanoma cells harboring *NRAS* mutations [94, 95]. *NRAS* mutations in melanoma promote RAS-MEK signaling cascade by switching their signaling from BRAF to CRAF, facilitated by the disruption of the cAMP-PKA inhibitory pathway on CRAF activity [94]. However, two similar reports have indicated that BRAF but not CRAF plays a critical role in initiating *NRAS*-driven melanoma, even though both display compensatory functions in tumor progression [96, 97]. Dorard et al. indicated that BRAF is crucial during the early stages of *NRAS*-driven melanoma [97]. Besides, BRAF and CRAF collaborate to activate ERK and maintain proliferation in *NRAS*-mutated human melanoma cell lines. Furthermore, under certain conditions, ARAF also emerges as a significant player. Notably, in the absence of both BRAF and CRAF, ARAF can promote cell proliferation. Similarly, depletion of both BRAF and CRAF has shown promising effects on *NRAS*^Q61L/K^ mutant melanoma cells [89]. Independent of its kinase activity, CRAF modulates tumorigenesis by inhibiting Rok-α activity within the CRAF-Rok-α complex, facilitating STAT3 phosphorylation, Myc expression, and tumor cell dedifferentiation [98]. In addition, CRAF is not necessary for ERK activation in promoting skin homeostasis [99]. This implies that if CRAF drives Ras-induced skin cancer through interactions with Rok-α or other substrates, therapeutic approaches would need to focus beyond merely inhibiting CRAF catalytic activity.

While the specific role of CRAF dimerization-dependent activation in *KRAS* mutant tumors is evident, it remains intriguing that not every *RAS*-mutated tumor relies on CRAF activation. Additionally, the kinase-dependent and -independent actions of CRAF vary across different tumor types. Given the significance of CRAF in *RAS* mutant tumors, there is potential for the rapid translational application of pan-RAF inhibitors, either alone or in combination with other targeted therapies.

### The role of CRAF in cancers with mutant BRAF

#### *BRAF*^V600^ mutant melanoma

The *BRAF*^V600E^ mutation, the most common BRAF genetic alteration, occurs in 66% of cutaneous melanomas and 25% of colorectal cancers [100, 101]. Generally, *BRAF*^V600E^ mutations cause sustained activation of the MAPK signaling pathway independent of the spatial activation and dimerization of RAF kinases. It has been observed that, compared to nevi tissues, melanomas exhibit elevated CRAF levels. Notably, depletion of CRAF levels compromises the viability of melanoma cells with either *BRAF*^V600K^ mutation or wide-type *BRAF* [102]. Although CRAF has been reported to be required for non-V600E BRAF melanoma cell viability through an allosteric conformation mechanism or direct phosphorylation of its activation segment, its function in BRAF^V600E^ melanoma is controversial [103–105]. Karreth et al. found that CRAF mRNA and protein levels in *BRAF*^V600E^ melanoma cells are lower than in cells harboring wild-type BRAF, suggesting that transcriptional regulation plays a vital role in the reduction of CRAF expression. One promising mechanism is that melanoma cells expressing BRAF^V600E^ bypass the antagonistic function of CRAF by reducing its expression. This, in turn, creates favorable conditions that promote MAPK pathway hyperactivation and cellular transformation [106].

While CRAF expression may differ in various types of *BRAF* mutant melanomas (*BRAF*^V600E^ or non-*BRAF*^V600E^), it is worth noting that increased CRAF levels have been reported to promote resistance in a subset of *BRAF* mutant melanomas [107]. CRAF overexpression and dysregulation are critical mechanisms for RAF inhibitor resistance in melanoma via reactivation of MAPK signaling [108]. Elevated CRAF expression can result in reduced primary drug sensitivity or acquired resistance to AZ628 (a selective RAF inhibitor) in *BRAF*-driven mutant cells. This phenomenon is associated with a target shift from BRAF to CRAF, a process in which the kinase activity of CRAF appears to be dispensable [107]. There is little doubt that *BRAF*^V600E^ mutations decrease their affinity with CRAF and the CRAF/BRAF ratio. Nevertheless, Karreth et al. discovered that CRAF elicited the inhibition of BRAF^V600E^ kinase activity and MAPK activation by forming BRAF^V600E^–CRAF complexes [106]. Under these circumstances, oncogenic RAS could influence the MAPK signaling cascade by augmenting the stability of the CRAF-BRAF^V600E^ complexes. The suppressive effect of CRAF on BRAF^V600E^ may indicate why oncogenic *RAS* mutations and *BRAF*^V600E^ have not been observed to occur concurrently. *BRAF*-mutant colorectal cancer is more prone to acquired resistance than *BRAF*-mutant melanoma, although CRAF was activated by oncogenic EGFR signaling in the former [109, 110]. Genetic ablation of *RAF1* increases the activity of BRAF and MAPK signaling in fibroblasts [111]. Another study indicates that selective CRAF inhibition promotes paradoxical activation, which indicates that CRAF may negatively modulate MAPK signaling in some instances [112]. It is noteworthy that clinical sample analyses have revealed the emergence of secondary benign and malignant skin tumors in *BRAF*^V600E^ melanoma patients undergoing BRAF inhibitor therapy. This phenomenon, linked to CRAF activation and BRAF-CRAF heterodimer formation, seems to be driven by *RAS* mutations. Specifically, oncogenic *RAS* mutations were detected in 58% of evaluated tumor samples (38/66) and 49% of control tumors from patients that had not received BRAFi therapy (30/62) [113]. These findings suggest a critical role for CRAF activation in acquired resistance to BRAF inhibitors in *BRAF*-driven tumors.

#### BRAF kinase-inactive mutation cancers

In addition to the dimerization-independent activation of *BRAF*^V600^, other *BRAF* mutants with impaired activity (also called class 3 *BRAF* mutants) were observed to stimulate MEK by alternatively activating CRAF via an allosteric or transphosphorylation mechanism [105]. Cytoplasmic mutant *BRAF*^G596R/G466V^ was found to activate CRAF via transphosphorylate of its activation segment and 14-3-3-mediated hetero-oligomerization in an *RAS*-independent manner [114]. This type of mutation enhances the binding of BRAF mutants to activated RAS, leading to the increased formation of heterodimers between mutant BRAF and wild-type CRAF [115]. Likewise, CRAF appropriates the signal from low-activity *BRAF*^G469E/D594G^ mutants and regulates apoptosis through mitochondrial localization via binding to Bcl-2 [116]. Moreover, ablation of CRAF suppresses MAPK signaling in cells harboring the impaired *BRAF* mutants but not *BRAF*^V600E^, which also indicates that sole inhibition of CRAF is not sufficient to abolish redundant activation of MEK by BRAF^V600E^. This finding suggests a critical role for CRAF kinase in enhancing resistance to BRAF inhibitors in *BRAF*-driven tumors. Additionally, certain inhibitory mutations in *BRAF* may result in CRAF assuming the mantle as the dominant driver of the MAPK signaling pathway. Heidorn et al. uncovered an intriguing phenomenon where kinase-dead BRAF (BRAF^D594A^) appears to necessitate the co-existence of oncogenic *RAS* to drive RAS-dependent CRAF activation and tumorigenesis [103]. This insight further highlights the connection between resistance to BRAF selective drugs and patients with *RAS* mutant tumors. However, BRAF^D594A^ was unable to activate CRAF or stimulate MEK phosphorylation, rendering it catalytically and biologically inactive [105, 117]. In summary, kinase-dead *BRAF* mutants, apart from *BRAF*^V600E^, may still activate MEK by inducing CRAF through diverse mechanisms. However, solely inhibiting CRAF may not fully halt MEK activation, underscoring the intricate connection between *BRAF* mutations and CRAF in modulating the MAPK signaling pathway.

### Key kinase-independent pathways of CRAF in cancers

While the kinase-dependent role of CRAF in the ERK-MAPK signaling pathway parallels that of BRAF, its kinase-independent function in oncogenic-driven cancers garners special attention. This unique role underpins the observed ability of prolonged CRAF ablation to prevent lung tumor initiation without inducing notable toxicity in adult mice. Such tumor regression likely arises from enhanced apoptosis rates combined with diminished cell proliferation. Yet, a clear understanding of the kinase-independent function of CRAF within *KRAS* and *BRAF* mutant tumors remains elusive. The therapeutic responses after CRAF suppression in lung cancer might be tied to the activation of pro-apoptotic pathways. In the following sections, we will dissect the central kinase-independent signaling pathways associated with CRAF (as depicted in Fig. 2).

p21-activated protein kinase-1 (PAK-1) was previously found to facilitate CRAF activation by direct phosphorylation of residues p.S338 and p.S339. The phosphorylated CRAF was subsequently translocated to the mitochondria and participated in protecting endothelial cells from intrinsic apoptosis in a kinase-independent manner [118, 119]. Further studies reveal that CRAF confers an anti-apoptotic effect when recruited to the mitochondrial membrane by Bcl-2. In contrast, CRAF recruited to the plasma membrane within the MAPK pathway does not manifest this anti-apoptotic effect [102, 120]. Based on this phenotype, Alavi et al. discovered that CRAF suppresses apoptosis by inhibiting stress-activated protein kinase ASK1, similar to the results observed by Chen et al. [121, 122]. Mammalian sterile 20-like kinase (MST2), a component of the Hippo signaling pathway was found to also contribute to the anti-apoptotic functions of CRAF independent of its kinase activity [123, 124]. Moreover, CRAF depletion promoted apoptosis by stimulating caspase-1 but not the MEK/ERK and NF-κB pathways [125]. Furthermore, the knock-down of CRAF inhibited the progression of *RAS*-driven and *BRAF*^V600K^ mutant melanoma by mediating the inhibition of Bcl-2 rather than by inhibiting the mitogen-activated protein kinase pathway [102]. Similarly, CRAF knockout was shown to suppress the proliferation of fibroblasts and hematopoietic cells by increasing the apoptotic index rather than through cell cycle disruption [111]. The aforementioned studies highlight the anti-apoptotic function of CRAF rather than its role in accelerating cell proliferation. Nevertheless, knock-down of CRAF prevented the phosphorylation of Bcl-2 and apoptosis induced by taxol [126]. Moreover, further studies indicated that phosphorylation of CRAF and Bcl-2, but not ERK1/2, was crucial in taxol-induced apoptosis in breast cancer cells [127]. Although the induction of apoptosis by taxol is dependent upon CRAF and Bcl-2 phosphorylation and Bcl-2 cleavage, the kinase activity of CRAF may be dispensable in this process.

CRAF promotes T helper cell differentiation and enhances immune responses by antagonizing Syk-induced RelB activation [128]. Furthermore, CRAF partially reprograms gene expression and regulates the cell cycle by activating the transcription of NF-κB through phosphorylation of IκB [129]. Moreover, CRAF was also shown to induce acetylation of the NF-κB p65 to modulate adaptive immunity by dendritic cells (DCs) [130]. However, the inhibition of RAF, but not MEK1/2, results in partial activation of CD4^+^ T cells during DC differentiation, suggesting that CRAF regulates DC function in a different manner than MEK1/2 kinase [131]. CRAF also plays a vital role in cancer cell proliferation by facilitating AURKA and Plk1 activation, mitotic spindle location, and tumor progression in a kinase-independent function [132]. Similarly, Advani et al. revealed that CRAF promotes DNA damage response and tumor radioresistance by elevating CHK2 activation through a kinase-independent mechanism [133]. Additionally, CRAF has been reported to antagonize the Rok-α kinase domain within its cysteine-rich regulatory domain, resulting in increased migration of keratinocytes/fibroblasts and tumorigenesis [134–136]. Furthermore, the disturbance of the CRAF-Rb interaction is sufficient to inhibit MMP-associated migration of cancer in vitro and in vivo [137].

## RAF1 alterations associated with cancer

### Aberrant expression of CRAF

Elevated CRAF protein expression is correlated with poor prognosis in hepatocellular carcinoma (HCC) patients treated with sorafenib [133]. Mutations in *RAF1* are extremely rare; however, overexpression of CRAF is correlated with disease progression in a subset of human cancers, including melanoma, non-small cell lung cancer (NSCLC), and hepatocellular carcinoma [102, 138, 139]. Overexpression of CRAF has been regarded as an early tumor marker for human lung adenocarcinoma [140]. Consistent with this observation, lung-restricted overexpression of full-length CRAF or its truncated kinase domain contributes to the MEK-dependent formation of lung adenomas [141, 142]. Moreover, increased CRAF levels have been reported to facilitate resistance in *BRAF* mutant melanomas [107]. However, the association between CRAF expression and tumor prognosis is controversial, and resistance mechanisms in vivo have not been demonstrated. According to the Gene Expression Profiling Interactive Analysis (GEPIA), an online tool for visualizing TCGA data, CRAF transcript expression does not completely align with the findings reported in the literature. For instance, CRAF expression in lung squamous cell carcinoma (LUSC) is significantly lower in tumors compared to normal tissues (http://gepia.cancer-pku.cn). Moreover, although CRAF overexpression is associated with tumor grade (*p* = 0.03), it appears that CRAF protein expression is not a reliable predictor of tumor progression [143].

Considering the intricate signaling biology of CRAF in the MAPK-dependent and MAPK-independent pathways, the diverse spectrum of alterations in CRAF and BRAF detected in cancer can manifest distinct functional attributes [82, 132]. Unlike *BRAF*, which is altered in up to 8% of all cancers, *RAF1* has a notably lower alteration frequency of 0.7% in cancers. This disparity could be attributed to its reduced basal kinase activity compared to BRAF and the necessity for more intricate regulatory processes for its activation [144, 145]. According to TCGA pan-cancer atlas results (https://www.cbioportal.org/), we identified that *RAF1* genetic mutations were present in 2.3% of all cancers. Specifically, *RAF1* mutations were frequently observed in skin cutaneous melanoma (5.41%, 24/444) and uterine corpus endometrial carcinoma (4.54%, 24/529), whereas *RAF1* amplification was highly concentrated in bladder tumors (10.71%, 44/411). Similar to Raie et al. [146], we observed that *RAF1* mutations or copy number alterations were rare (<3%) or absent in other tumor types. Overall, developing methods for treating *RAF1* mutant variants represent promising therapeutic targets in multiple cancer types.

### Point mutation of *RAF1*

Patients harboring the *BRAF*^V600E^ mutation have experienced clinical benefits from RAF inhibitors such as vemurafenib. However, with the observed limited median progression-free survival (less than 6 months) in melanoma treatments and the onset of rapid resistance, the focus has shifted to exploring combination therapy with MEK inhibitors [147]. Furthermore, numerous *RAF1* mutations that facilitate biochemical and pharmacological resistance have been identified (summarized in Table 1). By understanding *RAF1* mutations associated with drug resistance, we may enhance the likelihood of developing more effective therapeutic drugs [3, 7]. Demand for innovative treatments promotes the discovery of targetable chromosomal aberrances and mutations. For instance, a recent study demonstrated that *RAF1*^P261A^, located in the CR2 conserved region, promotes CRAF kinase activity in a dimer-dependent manner and benefits from the combination of LY3009120 and trametinib [4]. Another study demonstrated that *RAF1* p.S257 and p.S259 enhance oncogenic activity and sensitivity to sorafenib [6]. Moreover, researchers have identified single amino acid substitutions (p.S257P, p.P261T, p.G361A, p.E478K) within *RAF1* in melanoma cell lines resistant to RAF inhibitors [7]. In addition, Harms et al. identified a *RAF1*^G361A^ amino acid substitution in patients with Noonan syndrome that may be associated with a significantly higher incidence of hypertrophic cardiomyopathy (HCM) [5]. Likewise, *RAF1*^E478K^ mutation was found to constitutively heterodimerize and increase exogenous CRAF kinase activity. In contrast, another *RAF1*^R401H^ mutation was observed to impair basal CRAF activity and enhance the inhibition of CRAF kinase by RAF inhibitors [2]. The levels of phosphor-MEK1/2 correlate positively with the efficiency of B/CRAF heterodimer formation, which is impaired by *RAF1* interface mutations (p.E478K, p.R401H). Additionally, Atefi. M. identified a cancer-associated *RAF1*^R391W^ mutation in melanoma, which conferred vemurafenib-resistant MAPK pathway activation in a dimerization-dependent manner [3]. A screening trial consisting of 82 acute myeloid leukemia (AML) patients revealed that the CRAF p.S427G mutation, rather than the p.I448V mutation, triggers constitutive activation of ERK by activating the CRAF-ERK signaling cascade, even though both mutations are associated with ERK activation [8]. The observations gleaned from the aforementioned studies lead to a fundamental question: can membrane recruitment or CRAF kinase activity be impeded or abolished by a specific site mutation? Sanclemente M. et al. demonstrated that, despite both *RAF1*^D468A^ and *RAF1*^K375M^ mutations completely abolishing CRAF kinase activity, the phosphorylation states of CRAF p.S338 and p.S621 in these kinase-inactive isoforms were inversely affected. This suggests that the reduced phosphorylation of CRAF p.S338 and p.S621 might be attributed to conformational changes rather than impaired kinase activity [1]. Moreover, despite the suppression of CRAF kinase activity, the phosphorylation of MEK1 remained unaffected, further suggesting that CRAF kinase activity is not essential for the activation of the MAPK signaling pathway. Hatzivassiliou et al. also confirmed that kinase-dead *RAF1*^D486A^ was recruited to the plasma membrane in a kinase activity-independent manner [2]. Taken together, gain-of-function *RAF1* point mutations may contribute to paradoxical activation caused by the type I_1/2_ inhibitors through activated dimerization. Nevertheless, oncogenic mutant *RAF1* remains a rare target for the deployment of selective CRAF inhibitors in *RAF* or *RAS*-driven cancers.

### Gene fusion of *RAF1*

Oncogenic *RAF1* gene fusions have been observed in various cancers and RASopathies (summarized in Table 2). *RAF1* gene fusions commonly occur in pancreatic acinar cell carcinomas (up to 18.5% in all cases) [11]. Although melanomas with *RAF1* fusions are seldom observed (less than 1%), clinical sample analyses have consistently shown that melanomas harboring *RAF1* fusions exhibit wide-type status for *BRAF*, *RAS*, and *NF1* [12]. This finding implies that *RAF1* fusions could potentially serve as therapeutic targets in melanoma patients lacking *BRAF*^V600^ or *RAS* mutations. Additionally, the prevalence of certain gene mutations, including *TERTp* (62%), *CDKN2A* (60%), *TP53* (13%), *ARID2* (10%), and *PTEN* (10%), within melanomas with active *RAF1* fusions can aid in refining tumor classification strategies [12]. Phillips et al. corroborated this finding in anaplastic pleomorphic xanthoastrocytoma patients [20]. Two similar reports also described oncogenic *RAF1* rearrangement in pilocytic astrocytoma with elevated CRAF kinase activity and MEK phosphorylation [14, 15]. In a cohort study comprised of 7119 melanoma patients, 40 cases (0.6%) were identified with activated *RAF1* structural variants accompanied by mutations in *TERTp* and *CDKN2A* [12]. Another recent study identified a novel *LRRFIP2-RAF1* fusion in wild-type *BRAF* acral melanoma with a concomitant *KIT* variant [16]. Comprehensive genomic profiling (CGP) of 3,633 pediatric cancer patients revealed *RAF1* fusions in seven distinct pediatric tumor types. Within these fusions, *RAF1* was found to associate with several gene partners, including *MBNL1, TMF1, GOLGA4, SRGAP3, QKI, SOX6,* and *ATG7* [9]. Moreover, the *RAF1* fusion is also found in a specific molecular subtype in spindle cell tumors that co-express S100 and CD34 [17, 18]. Similarly, a striking case report identified a *MTAP–RAF1* gene fusion in an S100-positive soft tissue sarcoma [19].

Additionally, *RAF1* fusions can also facilitate MAPK pathway activation in multiple tumor types [15]. However, Jain et al. reported that *RAF1* fusions in pediatric low-grade gliomas (PLGGs) may not respond to type I and II RAF inhibitors previously proven effective in tumors harboring *BRAF* fusions [148]. The group also developed a heterologous *RAF1* fusion model and identified that the PLGGs are sensitive to pan-RAF and combinatorial inhibitors of the MAPK/PI3K signaling pathway. Clinical trials have indicated that *RAF1* gene fusions frequently occur in cases of acquired resistance to KRAS inhibitors (i.e., adagrasib and sotorasib); however, the underlying mechanisms contributing to this trend are currently unclear [149, 150]. Of note, results obtained from several preclinical studies investigating metastatic melanoma have indicated that activating *RAF1* fusions are sensitive to MEK inhibitors [10, 13, 151]. This evidence suggests that dimer-dependent activation of CRAF induced by *RAF1* fusions can be blocked by MEK inhibitors (i.e. selumetinib and trametinib). However, Jain et al. discovered that tumors with *RAF1* fusions only partially respond to MEK inhibitors [148]. Therefore, additional studies are needed to evaluate the efficacy of combination therapies that target RAF dimerization and MEK in malignancies harboring *RAF1* fusions.

Multiple studies have emphasized that the CRAF kinase domain–but not its kinase activity–plays a more pivotal role in *KRAS*-driven tumorigenesis [1, 74]. Therefore, other pathogenic oncogene fusions involving the CRAF kinase domain might serve as potential therapeutic targets. Research highlighting constitutive transformational activation of CRAF kinase induced by *RAF1* fusion with other truncated kinases may aid in identifying efficacious multi-target therapies.

### Other alterations of *RAF1*

*RAF1* amplifications, which enhance RAF/MEK/ERK signaling pathway activation, have recently been reported in various tumors. For instance, using data obtained from the GENIE v3 cohort, we observed that *RAF1* amplifications occur in bladder tumors at a frequency of 3.8% (139 out of 3844 patients), a rate higher than that for *RAF1* amplifications in any other tumor type within this cohort. As a result of *RAF1* amplification, bladder tumors with RAS oncogenic mutations are sensitive to RAF and MEK inhibitors.

Similarly, a randomized phase III clinical trial including 119 melanoma patients revealed that *RAF1* amplification elevated the efficacy of carboplatin and paclitaxel with sorafenib (CPS) in terms of progression-free survival (PFS) compared with carboplatin and paclitaxel treatment alone (CP) (HR, 0.372; *P* = 0.025) [152]. Additionally, a high level of *RAF1* amplification was observed in recurred/metastasized phyllode tumors of the breast compared with patients without recurrence/metastasis [153]. Coincidentally, a breast cancer study also indicated that dysregulation of the MAPK pathway due to *RAF1* amplification is associated with poor outcomes and resistance to PD-1/PD-L1 therapy. However, *RAF1* amplification is highly correlated with the genomically unstable (GU) Lund classification subtype, which responds best to the PD-L1 antibody atezolizumab, with approximately 50% of patients demonstrating a partial or complete response [154]. In terms of other rare genetic alterations, a recent report described a novel human truncated form of *RAF1* (*RAF1*-tr) that exhibited increased nuclear localization and enhanced the double-stranded DNA damage response through the modulation of PRKDC function in a RAS-MAPK independent manner [132]. In summary, CRAF amplification is associated with the activation of the RAF/MEK/ERK pathway.

## Molecular regulators of CRAF

### Upstream molecular regulation of CRAF

#### Epigenetic regulation of CRAF

miRNAs modulate target gene expression by interacting with the 3′-UTR region, resulting in mRNA degradation or inhibition of translation. Using luciferase reporter assays, researchers have identified a large category of miRNAs that interact with the 3′-UTR of *RAF1*, including miR-15a/b, miR-16, and miR-195. Consequently, processes such as cell proliferation, migration, senescence, and drug resistance are subject to modulation via miRNAs. Notably, miR-195 was found to significantly inhibit thyroid cancer cell proliferation by suppressing CRAF protein expression [155]. Evidence also suggests that miR-16 interacts with the 3′-UTR of *IGF1R*, *KRAS*, and *RAF1*, thereby reducing osteosarcoma cell proliferation through the CRAF–MAPK pathway [156]. Moreover, miR-424 was shown to trigger apoptosis and cell-cycle arrest in glioblastoma cells by directly targeting the *RAF1* and *AKT1* oncogenes [157]. Ghousein et al. also found that miR-4510 functions as a tumor suppressor in hepatocellular carcinoma (HCC) by directly targeting and inhibiting *RAF1* mRNA [158].

Moreover, ceRNAs can regulate CRAF expression through sequestering *RAF1* targeting miRNAs. LINC00460 was shown to enhance papillary thyroid cancer progression by targeting and neutralizing the suppression of miR-485-5p, a *RAF1-*targeting miRNA [159]. The lncRNA ITGB2-AS1 promoted pancreatic ductal adenocarcinoma progression by upregulating *RAF1* through sequestering miR-4319 [160]. Moreover, the LINC01559/miR-1343-3p/CRAF axis was found to promote pancreatic cancer progression [161]. ciRS-7, a potential miR-7 sponge, enhanced EGFR and CRAF activation, leading to a more aggressive colorectal cancer phenotype [162]. In addition, CircAGFG1/miR-370-3p and CircCDR1/miR-1287 were reported to regulate the transcription of *RAF1* in cervical cancer and hepatocellular carcinoma respectively [163, 164].

#### Regulation by transcription factors or transcription activators

A series of transcription factors regulate the transcription-mediated activation of the CRAF signaling pathway. AP-2α modulates the transcription of *RAF1* by amplifying its promoter transcriptional activity in HBV-expressing cells [165]. Similarly, bromodomain PHD finger transcription factor (BPTF) activates the MAPK pathway and is coexpressed with CRAF in T-cell lymphoma tissues [166]. Likewise, EZH2 contributes to impaired DNA damage repair and *RAF1* amplification. *RAF1* amplification leads to CRAF-β-catenin pathway activation and promotes stem cell self-renewal through the negative regulation of RAD51 [167]. Additionally, a report suggested that miR-493-3p inhibits RAF1 transcription by potentially decreasing the transcription of ETS1 [168]. Another study demonstrated that ETS2-mediated transcription of RAF1 promotes MAPK pathway activation [169].

#### Regulators of CRAF protein modification

Posttranslational regulation of CRAF is vital for CRAF stability and catalytic activity.

The RanBPM/CTLH complex promotes the ubiquitination and degradation of CRAF through its direct interaction with the C-terminus of CRAF [170]. Furthermore, CRAF protein stability is maintained by physical interaction with USP13, USP15 and inhibitors of apoptosis proteins (IAPs) at the post-translational level [171–173]. O-GlcNAcylation of CRAF promotes epithelial-mesenchymal transition (EMT) via inhibiting ubiquitination of CRAF, which is involved in the progression of renal interstitial fibrosis [174]. PRMT5 mediates the methylation of CRAF and promotes CRAF degradation and *RAS*-driven MAPK signaling [175]. Protein arginine N-methyltransferase 6 (PRMT6) inhibits aerobic glycolysis and cell stemness through the methylation of CRAF R100. This posttranslational modification of CRAF subsequently impedes PKM2 nuclear translocation and stem cell marker (CD133, SOX2, and NANOG) expression, respectively [176, 177].

#### Activating stimuli on CRAF

##### Scaffolding and chaperone proteins

In addition to being stimulated by the previously mentioned small GTP-RAS proteins, CRAF activity is also regulated by several MAPK scaffolding proteins, including KSR1/2, arrestins-2, SHOC2, 14-3-3 and PHB [67, 178–181]. The specific role of scaffolding proteins in a signal transduction cascade can vary depending on the specific target proteins involved. Unlike CRAF, KSR1/2 are characterized as pseudokinases owing to mutations in the active site. A previous report showed that KSR1 functions as an allosteric activator to promote CRAF catalytic function [182]. It is widely believed that KSR functions similarly to that of CRAF in the ERK pathway, as it competes with CRAF for binding to inhibited BRAF, resulting in allosteric activation [183]. In this context, KSR1 competes with CRAF for dimerization with BRAF in the presence of BRAF inhibitors. Given that CRAF-BRAF dimerization augments ERK signaling, KSR1 might effectively reduce the paradoxical activation of ERK signaling by promoting the complex formation between KSR and BRAF [184]. As a scaffold protein in the MAPK signaling cascade, arrestin-2 primarily interacts with CRAF but not MEK1 and ERK2 [178]. Additionally, β-arrestins promote phosphorylation of Src and thus enhance E2F expression driven by the CRAF-Rb complexes [185]. SHOC2 and the catalytic subunit of protein phosphatase 1 (PP1c) serve as highly specific effectors of M-Ras, critically influencing the activation of the MAPK pathway. Importantly, SHOC2 acts as a scaffold protein, mediating interactions between PP1C and M-Ras to specifically dephosphorylate the inhibitory p.S259 site on CRAF. This modulation enhances CRAF activity within distinct signaling complexes [186]. Doudican et al. found that the PHB1-CRAF complex mediates type I_1/2_ RAF inhibitor resistance; additionally, the group discovered that the conformational inhibitor rocaglamide A interrupts the interaction between PHB and CRAF, thus inhibiting the reactivation of MAPK signaling [108]. Most scaffolding proteins activate MAPK signaling by scaffolding kinase cascades; however, 14-3-3 constrains CRAF in an inactive conformation within the cytosol. Moreover, MAST1, another scaffold protein, contributes to cisplatin resistance by promoting CRAF-mediated activation of MEK, thereby exerting an anti-apoptotic effect [187]. Chaperone proteins, such as HSP90 and CDC37, play a role in maturing and moderating CRAF, subsequently facilitating mitogen-activated protein kinase pathway activation [59, 188]. Additionally, CNK1 regulates the activation of CRAF in a concentration-dependent manner by forming a trimeric complex with pre-activated CRAF and activated Src [189].

##### Other proteins

RIPK4 was shown to activate the CRAF-MEK-ERK pathway by promoting the degradation of proteasome-mediated phosphatidylethanolamine binding protein 1 (PEBP1) in pancreatic cancer [190]. PDK1 regulates P2Y receptor agonists-induced platelet activation via directly activating CRAF, which indicates that PDK1 regulates crosstalk between the canonical PI3K and MAPK pathways [191]. MAZ positively regulates CRAF signaling in pancreatic cancer by promoting PAK activation and AKT suppression through phosphorylation at p.S338 and dephosphorylation at p.S259, respectively. This regulation promotes epithelial-mesenchymal transition (EMT) [192]. In prostate cancer, PLK1 induces autophosphorylation at CRAF p.S621, which is crucial for protecting against degradation and regulating EMT and cellular motility [193, 194]. A study has shown that PHB1 and PHB2 interact with CRAF to facilitate chronic Hepatitis C Virus (HCV) infection. Notably, the group indicated that knock down of CRAF blocks HCV infection, whereas solely inhibiting RAF kinase fails to achieve the same outcome [195].

CRAF activity is indispensable in Lasonolide A (LSA) induced protein hyperphosphorylation and premature chromosome condensation independent of the MAPK pathway [196]. The activation of PAK1 and CHK2 is triggered through the p.S338 site of CRAF via a mechanism independent of its kinase activity [197]. It was reported that G protein-coupled receptors (GPCRs) activate CRAF through guanine nucleotide-binding G-proteins and the β-arrestins signaling pathway [198]. Interestingly, β-arrestins specifically bind to the Ras-binding domain of CRAF to balance CRAF activation due to stimuli from G-protein coupled receptors (GPCRs) and the EGFR-RAS signaling cascade. Several studies have reported that TM7SF2, PDCD6, p21-activated kinase (PAK3), and serine/threonine kinase 3 (STK3) contribute to tumorigenesis via direct binding and activation of CRAF [199, 200]. It was also reported that CRAF-ERK is the dominant pathway involved in HER-2-mediated tumor progression [201]. As a complex regulator of the MAPK signaling cascade, PP2A positively regulates this pathway by catalyzing the dephosphorylation CRAF Ser259 [202].

#### Negative regulation of CRAF

The unexpected regulation of MAPK signaling by cAMP/PKA is partly due to Rap1-mediated suppression of CRAF [203]. Although Rap1 activates ERK signaling through BRAF, the overall effect of cAMP/PKA on this pathway is determined by the ratio of CRAF, BRAF, and PKA isoforms [204–206]. A study has reported that CRAF reverts to a signaling-competent state through interactions with protein phosphatase PP2A and prolyl isomerase Pin1. PP2A dephosphorylates CRAF, while Pin1 catalyzes the isomerization of its phosphorylated residues. This process facilitates the efficient recycling of CRAF within the MAPK/ERK signaling pathway [207]. The cross-talk between the PI3K-AKT and RAF-MAPK pathways in cell proliferation, metabolism, and motility is apparent due to the interaction between AKT and CRAF. It has been observed that AKT (also known as protein kinase B) suppresses CRAF at the S259 site, resulting in cross-inhibition between the AKT and ERK pathways [208, 209]. Serine/threonine protein phosphatase 5 (PP5) is traditionally thought to negatively regulate MAPK signaling by dephosphorylating CRAF at p.S338. However, a study by Matthew et al. recently shed light on a potentially contradictory role of PP5. Their work indicates that PP5's influence on CRAF's feedback phosphorylation is also contingent upon forming PP5-ERK1/2 complexes, a process driven by active Rac1 [210]. By binding to the N-terminal region of CRAF, the CRAF kinase inhibitor protein (RKIP) negatively regulates CRAF, a process vital for cell growth and differentiation [211, 212]. A previous study showed that the RKIP inhibitor suramin enhances the MAPK pathway by preventing RKIP from binding to CRAF [213]. EphA2 inhibitor dasatinib interferes with the BRAF/CRAF heterodimer activity via elevating caveolin-1 (CAV-1) in uterine carcinoma [214]. Another report reveals that PHLPP1/2 dephosphorylates CRAF, diminishing colorectal cancer cell invasion and migration [215]. SPRY2 attenuates B-cell receptor (BCR) and MAPK-ERK signaling by binding to CRAF and BRAF in normal B cells and chronic lymphocytic leukemia (CLL) cells [216].

### Downstream effectors of CRAF

#### Catalytic effects of RAF kinase on MEK

Under physiological conditions, *RAS*-driven activation of RAF proteins occurs on the plasma membrane where activated RAS promotes RAF dimerization, a pivotal event to trigger the kinase activity of RAF proteins. The observation that kinase-dead BRAF was able to activate ERK signaling through dimerizing with and activating CRAF provided further support for the role of RAF dimerization and raised awareness that catalysis-dependent and -independent functions of RAF are functionally important [103, 217, 218]. In addition to the BRAF-CRAF heterodimer, respective homodimers of the two isoforms have also been detected but were noted to exhibit lower kinase activity. Notably, RAF family members can form physiologically relevant heterodimers and homodimers, resulting in their transactivation [180, 219]. Once RAF adopts an active conformation, its dimer interface is further stabilized by the hydrophobic Rspine residue in the αC-helix (p.L505 for BRAF, p.L397 for CRAF, and p.L358 for ARAF) located adjacent to the conserved RKTR motif [220]. Upon the relocation of R509 to the center of the dimer interface, αC-helix interacts with the NTA motif of the trans-RAF molecule and adopts the “IN” conformation [221, 222].

RAF phosphorylates MEK p.S218 and p.S222 within the activation loop; however, this activation also necessitates the prior association of MEK with RAF [223]. In a quiescent state, BRAF and MEK coalesce to form a heterodimer within the cytosol. Under these conditions, CRAF and ARAF abstain from interactions with MEK, prompting questions about their recruitment strategies for MEK [224]. Protein crystallography studies have indicated that BRAF directly interacts with MEK1, establishing contact predominantly through the αG helices and the activation loop. Concurrently, RAF proteins showcase a propensity to self-dimerize in a side-to-side fashion [223]. Studies have highlighted that RAF dimers or the homodimer of MEK itself predominantly phosphorylate the MEK homodimer but not its monomeric counterpart [225, 226]. During the activation phase, RAF and MEK collaboratively form a tetrameric complex, illustrated as MEK-RAF-RAF-MEK in crystallographic studies [100]. Subsequently, the phosphorylated MEK activates ERK.

Beyond the four standard components of the Ras-Raf-MEK-ERK signaling pathway, KSR1/2 serves as a pivotal scaffolding protein, facilitating the assembly of Raf-MEK-ERK complexes. Brennan and colleagues observed that BRAF enhances KSR2 activity by forming BRAF-KSR2 heterodimers, subsequently promoting the phosphorylation of MEK1 [224]. Notably, Lavoie et al. discovered that MEK facilitates the side-to-side dimerization of the BRAF–KSR1 kinase domain independently of MEK's catalytic activity [227].

#### CRAF's non-catalytic target regulation

Activated CRAF was previously reported as a potential therapeutic target against immune escape via stimulating TLR4-mediated inflammatory responses [228]. Moreover, it has been observed that the interaction between CRAF, Aurora-A, and Plk-1 at the centrosomes and spindle poles plays a pivotal role in promoting mitosis. Similarly, allosteric inhibitors of CRAF, but not ATP-competitive inhibitors, induce G2/M phase arrest by impairing the activation of Plk1 [132]. Through suppressing the pro-apoptotic kinases BAD [229], ASK1 [122], and MST2 [123], CRAF exerts a significant influence on apoptosis in a MAPK-independent manner. Another report reveals that crosstalk between the MAPK and Hippo signaling pathways depends on the CRAF/MST-2 complex [230]. Furthermore, CRAF facilitates the recruitment of Rokα, a function intriguingly not reliant on its kinase activity [231]. Direct inhibition of Rokα-mediated keratinocyte dedifferentiation by blocking CRAF prevented GDC-0879 induced tumorigenesis [136, 232]. Furthermore, CRAF was found to promote cell proliferation and migration in human lung fibroblasts through the TGF-β1/CRAF/Smad pathway [233]. A previously published report indicated that CRAF promotes the transformation of fibroblast cells through MEKK1-mediated NF-kB activation [234]. Upstream and downstream molecular regulation of CRAF across different cancer hallmarks has been summarized in Fig. 3.

## Combination therapy and related anti-tumor applications

### Strategies for CRAF inhibition

Since the discovery of oncogenic *RAF1*, there has been a concerted effort to develop therapeutic inhibitors to attenuate its aberrant activity in tumor cells. Recent studies have confirmed CRAF as a promising therapeutic target in *KRAS*-driven NSCLC [80]. Depletion of CRAF has been shown to decrease tumor size without notably affecting MAPK signaling in *KRAS*-driven lung cancer [83]. These findings have spurred a strong interest in selectively targeting CRAF as a potential treatment for *KRAS* mutant lung cancer. Current research indicates that CRAF has diverse functions in cancer, encompassing both kinase-dependent and kinase-independent mechanisms. The absence of significant toxicity upon CRAF depletion suggests that its primary mode of action might extend beyond the MAPK signaling pathway. Additionally, given the marked structural and functional similarities between BRAF and CRAF proteins, devising specific CRAF kinase inhibitors is inherently challenging. However, there still exist potential strategies worthy of exploration.

#### Inhibitors for selective CRAF kinase

Sorafenib, designed initially as a CRAF kinase inhibitor, has shown limited efficacy in clinical trials for melanoma, with favorable clinical responses less than 5% [235]. While sorafenib inhibits CRAF, wild-type BRAF, and BRAF^V600E^ kinases, it also targets other kinases such as Flt3, Kit, and VEGFR. A series of selective CRAF inhibitors have been successfully developed in vitro by modifying the structure of existing BRAF and pan-RAF inhibitors, enabling them to specifically target CRAF. The selective CRAF inhibitor ZM336372 significantly reduces bioactive hormone levels and human achaete-scute homologue-1 (ASH-1) expression in carcinoid tumor cells, leading to pronounced suppression of cellular proliferation and the cell cycle [25]. Recently, Zhao et al. identified a novel spirocyclic CRAF inhibitor, SHR902275, which has exhibited excellent drug metabolism and pharmacokinetic properties in vivo [26]. GW5074, a CRAF inhibitor, was found to enhance the anticancer effects of sorafenib by inducing mitochondrial dysfunction [22, 23]. The pyrimidin-4-yl-1H-imidazol-2-yl derivative 7a showed potent and selective inhibition of CRAF with an IC_50_ value of 0.62 μM and demonstrated superior antiproliferative activity compared to Sorafenib [29]. Several compounds have been reported as highly potent and selective CRAF inhibitors, including (4-aminobenzyl/benzoyl)-1H-imidazol-1-yl pyrimidin-2-yl derivatives 10c, with an IC_50_ of 8.79 nM [28]. Other promising compounds include 1,4-dihydropyrazolo [4,3-d]imidazole phenyl derivatives 2t, with IC_50_ values ranging from 0.56 to 0.86 μM in WM3629 cell lines [27], and pyrimidin-4-yl-1H-imidazol-2-yl derivatives 7a, showing IC_50_ values of 0.62 and 4.49 μM in A375P and WM3629 cell lines respectively [29]. 3-carboxamido-2Hindazole-6-arylamide 10d is also a potent CRAF inhibitor, which exhibits an IC_50_ of 38.6 nM [30]. Various natural small molecules have also been identified as selective inhibitors of CRAF. One such example is gallic acid, which inhibits MMP-1 expression through targeting CRAF [236]. Luteolin, a natural CRAF inhibitor, reduces inflammatory responses in human neutrophils by inhibiting the MAPK signaling pathway [237]. Another natural compound, erianin, the main component of Dendrobium chrysotoxum, has been found to inhibit the progression of melanoma and colorectal cancer by targeting CRAF and downstream MEK1/2 [50]. In a previous study, researchers have demonstrated that the targeted delivery of mutant *RAF1* to the neovasculature using nanocrystals exhibited anti-angiogenic effects. These findings suggest novel prospects for targeting tumor neovasculature with small-molecule drugs that act specifically on CRAF. Such targeted interventions may induce apoptosis in endothelial cells and lead to regression of tumor vasculature [118, 238]. Despite being developed as specific CRAF inhibitors, many compounds still exert inhibitory effects on BRAF kinase due to the highly homologous protein structures of B/CRAF.

#### Inhibitors for the scaffold proteins or partners of CRAF

Scaffolding proteins are central to orchestrating MAPK pathway activity. MAPK scaffold proteins notably (i) connect directly with various MAPK signaling components, (ii) coordinate or segregate protein interactions, and (iii) modulate signal intensity to specific stimuli, ensuring precise and timely MAPK signal relay. Importantly, therapeutic strategies are available to target scaffolding and chaperone proteins that interact with CRAF. Scaffold protein HSP90 was reported vital for CRAF activation via dephosphorylation of the p.S259 residue. KBU2046 selectively inhibits the activation of CRAF and modulates cell motility by binding to the interface of HSP90/CDC37, thereby disturbing the interaction between the CRAF and HSP90/CDC37 heterocomplex [33]. It has been reported that radicicol and novobiocin induce the degradation of the HSP90 client protein CRAF but do not degrade BRAF^V600E^ or inhibit MEK1/2 activation in HT29 human colon cancer cells [34]. Peptide R18 is found to effectively block the interaction between CRAF and the physiological ligand of 14-3-3, thereby inhibiting the protective effect of 14-3-3 against phosphatase-induced inactivation of CRAF [239]. RKIP has been reported to inhibit the phosphorylation of CRAF at S338 and Y341. Additionally, small molecule ligands such as DHPE and Locostatin interfere with the interaction between CRAF kinase and RKIP [35]. Moreover, suramin directly binds to RKIP and prevents its inhibitory effect on the MAPK signal pathway [213]. These scaffolding proteins, when bound to CRAF, influence the activation of the MAPK cascade and also facilitate CRAF degradation. However, due to the nonspecific nature of client proteins, inhibitors might counteract oncogene switching, a key mechanism by which tumors evade kinase inhibitors [240].

#### Inhibitors for the upstream and downstream protein of CRAF

##### Inhibitors for KRAS

As essential upstream regulators of CRAF, members of the RAS family of GTPases, which include KRAS, NRAS, and HRAS, undergo a transition between the GTP-loaded “on” state and the GDP-loaded “off” state. This transition is orchestrated through the activity of RAS guanine nucleotide exchange factors (RAS-GEFs) and RAS GTPase-activating proteins (RAS-GAPs), respectively [241, 242]. Considerable efforts have been devoted to suppressing RAS oncogenic signals by addressing upstream proteins, downstream proteins, and directly targeting RAS itself. During the activation-inactivation process, the importance of conformational alterations in two specific regions of the RAS protein, notably in switch II, has become apparent and has played a pivotal role in the eventual progression of RAS inhibitors [243]. Efforts are currently in progress to create mutant-specific RAS inhibitors that target *KRAS*^G12C^ in the switch-II region. The approval of Sotorasib for treating *KRAS*^G12C^ NSCLC represents a noteworthy achievement as the initial targeted therapy for tumors harboring *KRAS* mutations, offering hopeful prospects for the advancement of similar allele-specific treatments for mutant *RAS* [244]. Several potent covalent inhibitors of KRAS^G12C^ acting through a similar mechanism have entered clinical development. Adagrasib has demonstrated a significant reduction in cellular viability exclusively in *KRAS*^G12C^ cell lines and induced tumor regression in xenograft models [92]. Notably, Adagrasib has received FDA approval for the treatment of previously treated advanced-stage *KRAS*^G12C^ mutant NSCLC, based on the results of a phase I/II clinical trial (NCT03785249). Furthermore, both monotherapy and combination therapies involving covalent irreversible *KRAS*^G12C^ inhibitors, in conjunction with other targeted agents, are currently undergoing clinical trials for patients with advanced-stage *KRAS*^G12C^ mutant solid tumors. Examples include GDC-6036 in combination with the SHP2 inhibitor GDC-1971 (NCT04449874), JDQ443 in combination with another SHP2 inhibitor TNO155 (NCT04699188), and LY3537982 in combination with the CDK4/6 inhibitor Abemaciclib or the PD-1 inhibitor Pembrolizumab (NCT04956640).

Mutations in RAS proteins also influence their binding affinity with downstream effectors. For instance, KRAS^G12D^ exhibits a notably fivefold weaker binding to the CRAF-RBD compared to wild-type KRAS [245]. Thus alternative therapeutic strategies exist to inhibit CRAF activity, such as targeting its interaction with upstream KRAS oncoproteins. By disrupting the interaction between RAS and its downstream effector CRAF, B4-27 has demonstrated potent inhibition of RAS signaling in RAS-mutant cancer cells [246]. The compounds Kobe0065 and Kobe2602 have been identified as potential inhibitors that interrupt the binding between HRAS and CRAF. Furthermore, the compounds effectively suppressed the activity of the kinases located downstream of MEK at a concentration of 20 μM in *HRAS*^G12V^ mutant NIH3T3 cells [31]. MCP110 effectively blocks RAS-induced activation of CRAF in vitro, resulting in reduced anchorage-independent cell growth, the induction of G1 cell cycle arrest, and decreased cyclin D expression in A549 cells [32]. Moreover, rigosertib has been characterized as a RAS mimetic compound with the ability to disrupt the interaction between the RAF and PI3K protein families with KRAS [247].

##### Inhibitors for downstream apoptotic effectors

Indeed, although targeting the interaction between CRAF and its upstream activator KRAS can be a promising strategy, selectivity remains a pressing concern. A more selective approach can be achieved by targeting the specific interaction between CRAF and its apoptotic effectors, such as ROK-α, ASK1, and MST2, which operate independently of its kinase activity [99, 122, 124]. Nevertheless, further efforts are required to fully validate the therapeutic potential of targeting CRAF effectors. Structural studies and mapping of protein-protein interaction interfaces can provide valuable insights into the molecular mechanisms underlying these interactions.

### pan-RAF inhibitor therapy

In the past few decades, the discovery of *BRAF*^V600E^ mutations, which are oncogenic and highly active in most melanomas, has spurred significant interest in targeting this particular kinase [248, 249]. Type I_1/2_ inhibitors (Fig. 4), including Vemurafenib and Dabrafenib, selectively associate with the "active" DFG-in and αC-helix-out conformation of the ATP binding site, thereby specifically targeting BRAF^V600E^ [250]. However, these RAF inhibitors often paradoxically activate the MAPK signaling pathway in *RAS*-driven tumors by promoting dimerization of inhibited BRAF with CRAF. To overcome the activation of RAF homo- and heterodimers, further development of type II pan-RAF inhibitors able to bind with the "inactive" DFG-out and αC-helix-in conformation at the ATP binding site has been pursued [251]. These inhibitors exhibit comparable potencies in stabilizing the αC-helix-in conformation of RAF proteins, effectively targeting both active RAF dimers and monomers. However, due to their similar potencies in targeting BRAF and CRAF, the process of transactivation between dimer partners is minimized [252].

Taking these findings into consideration, a class of RAF inhibitors, which utilize the structure of Type I_1/2_ inhibitors and are referred to as paradox breakers, such as PLX8394, have been developed. These inhibitors counteract the paradoxical activation of ERK by targeting BRAF-containing dimers, while preserving RAF function in normal cells where CRAF homodimers facilitate signaling, and selectively disrupt RAS-independent BRAF-driven signaling. Concerning the mechanism, these inhibitors demonstrate a stronger affinity for both BRAF homodimers and BRAF-CRAF heterodimers, yet they are less effective against CRAF, potentially suggesting reduced impact on *KRAS*-driven cancers [253]. In addition, several "type II" RAF inhibitors, including TAK580, TAK632, LHX254, BGB283, and RAF709, have been developed as potent inhibitors of RAF dimer activity. By displaying similar activity against monomeric and dimeric forms of RAF and minimizing off-target activation of wild-type *RAF*, these inhibitors have proven effective in blocking MAPK signaling in tumors harboring *BRAF* or *RAS* mutations [36–38, 254–256]. LY3009120, another pan-RAF inhibitor, preferentially inhibits the kinase activity of RAF dimers [257]. Additional selective RAF dimer inhibitors such as belvarafenib (GDC-5573) have shown preliminary efficacy in *BRAF*^V600E^ and *RAS*-mutated advanced solid tumors in the early clinical phases. According to these findings, the development of DFG-out-type pan-RAF inhibitors holds greater potential for treating patients with cancers carrying oncogenic *BRAF*^V600E^ or *NRAS* mutations. Moreover, several DFG-out type pan-RAF inhibitors (such as RAF265, TAK632, and LY3009120) have entered clinical research programs [40, 44]. Interestingly, *ARAF* mutations were shown to promote resistance to belvarafenib through dimerization-dependent and kinase activity-dependent mechanisms [42]. It is important to note that although pan-RAF inhibitors have shown promising efficacy in vitro, non-specificity for BRAF mutations could also suppress wild-type RAF dimer activity in normal cells [40, 258]. Additionally, the inherent limitation of target-based therapy lies in the narrow specificity of the agents used, which can be circumvented by activating alternative survival pathways in cancer cells. This concept is based on the understanding that survival pathways have pleiotropic effects. Over time, cancer cells have evolved within the host, enabling them to activate multiple signaling pathways to evade apoptosis and promote proliferation. Consequently, utilizing multi-target agents presents a promising approach to overcome these limitations.

### Resistance mechanisms to RAF inhibitor

#### Resistance mechanisms in cancer with mutant BRAF

Recent studies have identified multiple primary mechanisms of resistance to RAF inhibitors in BRAF^V600E^ melanoma. Lito et al. reported that cancers with *BRAF*^V600E^ mutations develop resistance to I_1/2_ RAF inhibitors primarily through elevating active RAS-GTP levels and altering *BRAF*^V600E^ splicing [259]. Alterations in *BRAF*^V600E^ splicing variants without N terminus (V600E/ΔNT) and BRAF(ΔVNTAP) were discovered to promote the formation of protein homodimers and diminish the efficacy of the type I_1/2_ and II RAF inhibitors [225, 260, 261]. Additionally, several studies suggest that diverse oncogenic modifications stabilize the R-spine of BRAF, leading to constitutively active kinases resistant to RAF inhibitors [221, 225, 262]. Yap et al. found that dimer affinity is not directly tied to drug resistance in *BRAF* mutant cancers [263]. The group also observed that the enhanced stability of the R-spine in *BRAF* mutants with LLR^ins506^/VLR^ins506^ insertions drives resistance to both types I_1/2_ and II RAF inhibitors. Interestingly, these specific mutations significantly decrease the dimerization of oncogenic *BRAF* mutants. Intriguingly, drug-resistant cells become dependent on RAF inhibitors, and discontinuing the treatment slows the growth of resistant tumors [264].

Another avenue involves the activation of parallel (or "bypass") signaling pathways. For instance, the diminished efficacy of the I_1/2_ inhibitor in BRAF-mutated CRC is mostly linked to enhanced MAP kinase pathway-independent mechanisms involving EGFR signaling or the PTEN-PI3K-AKT signaling axis [109, 265, 266]. Interestingly, under basal conditions, RAF-MAPK signaling inhibits RTK–EGFR signaling via a negative feedback loop [110]. Moreover, several oncogenic mutations contribute to acquired resistance against BRAF inhibitors. Specifically, even after administering BRAF inhibitors, activating mutations in *RAS* and *MAP2K1/2* can still reactivate the MAPK kinase pathway [267–269]. Furthermore, acquired resistance can also arise from the reactivation of MAPK signaling due to CRAF overexpression/mutation and COT overexpression [7, 270]. Studies have pinpointed elevated CRAF protein levels in melanoma cell culture models as a potential mechanism for BRAF inhibitor resistance [107]. COT diminishes the sensitivity of BRAF^V600E^ melanoma cells to vemurafenib via MEK, bypassing the RAF signaling [271]. Dimerization of BRAF and CRAF results in increased accumulation of nuclear β-catenin in cancer-associated fibroblasts (CAFs), which further contributes to resistance against BRAF inhibitors [272]. In light of these insights, delving deeper into the clinical significance of increased CRAF protein levels becomes crucial when addressing BRAF inhibitor resistance.

#### Resistance mechanisms in cancer with wild-type BRAF

In wild-type *BRAF* isoforms, RAF inhibitor-induced paradoxical activation arises due to enhanced dimerization of BRAF and CRAF [2, 105]. One mechanism is that the inhibitor binding to CRAF facilitates the formation of CRAF homodimers, activating CRAF and triggering downstream MEK-ERK activation. Another potential, yet nonconflicting, mechanism involves the inhibitor binding to BRAF, resulting in a BRAF-CRAF heterodimer and subsequent CRAF activation. Correspondingly, pan-RAF inhibitors effectively target both protomers of the RAF dimers. Meanwhile, upon binding, paradox breakers induce a transition to the αC-helix out conformation that blocks dimerization-driven transactivation. Deepening our understanding of these inhibitor-RAF interactions and the reactions of wild-type RAF can pave the way for reducing off-target effects in patients.

### Combination therapy of pan-RAF inhibitors

Until now, type I_1/2_ RAF inhibitors have exhibited limited efficacy when utilized in the context of colorectal and thyroid tumors harboring *BRAF* mutations [100, 273]. With advances in cancer treatment, single therapeutic strategies no longer suffice in effectively tackling the complex and diverse mechanisms that drive tumor growth and progression. Therefore, combination therapy is considered a more effective cancer treatment strategy as it targets multiple molecular targets simultaneously. For instance, pan-RAF inhibitor LXH254 blocks dimeric BRAF and CRAF, which can provide a potential clinical strategy when combined with MEK or ERK inhibitors to treat *KRAS* mutant NSCLC or *NRAS* mutant melanoma [38]. In addition, EGFR-mediated activation of RAS and RAF serves as the impetus for the reactivation of MAPK signaling in a subset of *BRAF*-mutant CRCs [109, 110]. The findings emphasize the value of combining RAF inhibitors with immune checkpoint inhibitors, tyrosine kinase inhibitors, or MEK inhibitors to enhance clinical outcomes and delay drug resistance [274].

#### Combination of MEK and pan-RAF inhibitors

It is important to note that the efficacy of therapeutic strategies relying on CRAF inhibition has been limited to inducing complete tumor regression in a small proportion of cases. Consequently, the successful development of clinically effective therapies targeting KRAS-mutant tumors may require the discovery of potent inhibitors targeting CRAF and the identification of additional targets to expand the spectrum of responsive tumors.

A phase I trial (NCT02407509) revealed that the novel MEK-RAF inhibitor, CH5126766, demonstrated significant efficacy in treating solid tumors and multiple myeloma with MAPK pathway mutations, with 27% of the 26 assessed patients achieving objective responses [46]. Erianin, a MEK-CRAF inhibitor, was reported to suppress the constitutive activation of the MAPK signaling pathway and exhibit anti-tumor effects in melanoma and colorectal cancer PDX models [50]. RAF709 has demonstrated superior antitumor activity in cell line and tumor xenograft models with *BRAF* or *RAS* mutations. Moreover, when combined with MEK inhibitor trametinib, RAF709 produced a heightened antitumor response in *RAS*-mutant models compared to RAF709 treatment alone [254]. It was previously shown that the selective pan-RAF inhibitor TAK-632 exhibits synergistic effects with the MEK inhibitor TAK-733 in BRAF inhibitor-resistant melanoma [37]. In a Phase Ib upgrade/expansion study, LXH254 was evaluated in conjunction with trametinib among patients grappling with advanced/metastatic non-small cell lung cancer harboring *KRAS* or *BRAF* mutations and *NRAS-*mutated melanoma (NCT02974725). Importantly, LXH254 produced promising preliminary antitumor efficacy in *NRAS*-mutated melanoma patients [39]. Additionally, the synergistic modulation of the MAPK pathway was identified in an HCT116 xenograft mouse model upon co-administration of GNE-9815 (or GNE-0749) and cobimetinib [275, 276].

Specific *ARAF* mutations may contribute to acquired resistance to RAF dimer inhibitors, such as belvarafenib. A promising clinical strategy is to combine RAF and MEK inhibitors, as demonstrated in ongoing clinical trials (NCT02405065, NCT03118817), which was recently proposed to delay the onset of *ARAF*-driven resistance [42]. Based on structural analysis, belvarafenib and GW5074 may serve as promising templates for developing covalent inhibitors that selectively target pan-RAF or CRAF. Notably, cobimetinib, a type III MEK inhibitor, holds the potential for modification to selectively inhibit the MEK1/2 allosteric activity. These insights offer a basis for medicinal chemists to design novel covalent inhibitors for targeting the MAPK pathway [277].

#### Combination of EGFR tyrosine kinase inhibitors and pan-RAF inhibitors

Temporary suppression of phospho-ERK through the use of BRAF inhibitors is seen in CRCs harboring *BRAF* mutations. However, a reactivation of ERK takes place due to EGFR-mediated activation of RAS and CRAF. Interestingly, *BRAF* mutant CRCs exhibit elevated levels of phosphor-EGFR compared to *BRAF* mutant melanomas. This phenomenon suggests that CRCs are particularly inclined towards developing EGFR-mediated drug resistance. Concurrent inhibition of RAF and EGFR has demonstrated the ability to prevent the reactivation of MAPK signaling in *BRAF* mutant CRC cells [109]. Likewise, the simultaneous suppression of CRAF and EGFR expression, which is crucial for the onset of pancreatic metaplasia, effectively halted tumorigenesis [278, 279]. Of greater significance, simultaneous excision of CRAF and EGFR alleles in mice with existing tumors resulted in complete tumor regression in a subset of the mice. However, the exact mechanism by which EGFR ablation collaborates with the loss of CRAF expression to achieve this outcome is not fully understood. Importantly, when CRAF and EGFR were simultaneously targeted, no additional toxicities were observed beyond the skin alterations [85]. As previously mentioned, studies have indicated that acquired resistance to the RAF inhibitor PLX8394 occurs through EGFR-mediated RAS-mTOR signaling. However, early combination therapy of PLX8394 with EGFR or mTOR inhibitors can prevent resistance to PLX8394. These findings provide a sound biological rationale and a potential combinatorial treatment strategy to facilitate the application of PLX8394 in *BRAF* mutant lung cancer patients [280]. In addition, results from a phase I study have demonstrated that Lifirafenib, a newly developed inhibitor targeting RAF and EGFR kinases, exhibited a favorable risk-benefit profile and has shown antitumor activity in patients with solid tumors harboring *BRAF*^V600^ mutations [281].

#### Combination of immune checkpoint inhibitors and pan-RAF inhibitors

In recent years, increasing evidence has suggested that combining immune checkpoint inhibitors (ICIs) and BRAF/MEK inhibitors can enhance the efficacy of cancer treatment, especially for patients who are resistant to monotherapy of ICIs. Similarly, clinical trials (NCT02224781) have demonstrated that sequential use of immune therapy (Ipilimumab and Nivolumab) followed by BRAF-targeted treatment (Dabrafenib and Cobimetinib) achieves superior therapeutic effects compared to the reverse sequence [282]. However, the combination of pan-RAF inhibitors and immune checkpoint inhibitors is still in the early exploratory stage.

An ongoing phase I clinical trial is actively exploring the therapeutic dose of oral pan-RAF inhibitor LXH254 in combination with PDR001 PD-1 monoclonal antibody in patients with advanced solid tumors (including NSCLC, ovarian cancer, and melanoma) with MAPK pathway alterations [283]. Furthermore, a phase Ib clinical trial evaluating the combination of regorafenib and nivolumab demonstrated that the aforementioned dual therapy has a manageable safety profile and exhibits promising antitumor activity [47]. Overall, the combination of pan-RAF and immune checkpoint inhibitors has produced promising results in preclinical and clinical studies, offering new therapeutic options for patients with various solid tumors. Further studies are warranted to optimize treatment regimens and identify patient populations that can benefit the most.

#### Combination therapy of pan-RAF inhibitors with other inhibitors

Dysregulation of MAPK and PI3K/Akt signaling pathways plays a critical role in the pathogenesis and progression of various cancers, particularly those driven by oncogenic *RAS* mutations. The therapeutic efficacy of CDK4 kinase inhibition is restricted in the treatment of *KRAS*-driven lung adenocarcinomas; however, an intriguing approach involving the combination of CDK4 kinase inhibition and elimination of CRAF expression has demonstrated acceptable toxicities in preclinical in vivo studies [81]. Disrupting the interaction between Rb and CRAF by RRD-251 was found to substantially reduce the malignant characteristics of pancreatic cancer cells, regardless of their sensitivity to gemcitabine [284]. A combination index analysis revealed a notable synergistic effect of the RAF265/SB590885 + ZSTK474 treatment regimen in papillary thyroid cancer cell lines [45]. The combination of AZ628 and BP-1-102 significantly suppressed MEK/ERK signaling pathway activation in lung cancer cells harboring *KRAS* mutations. This suggests that a combination of pan-RAF and STAT3 inhibitors could be an effective treatment for lung cancer cells with *KRAS* mutations [285]. Another study illustrated that combining pan-RAF (RAF265) and mTOR inhibitors (RAD001) enhanced the anti-tumor effects through the RAS-RAF and PI3K pathways, possibly through targeting the 4EBP1 and S6 protein [286]. The combination of RAF265 and BEZ 235 (a PI3K inhibitor) significantly inhibited the growth of xenograft tumors with *KRAS* and *RET* mutations, suggesting that blocking the ERK and PI3K signaling pathways can effectively inhibit tumor progression in differentiated and medullary thyroid cancer [287]. Our previous research indicated that TOPK activates the AKT/mTOR signal pathway and ERK signaling pathways in different esophageal cancers, suggesting potential opportunities for combination therapies with TOPK inhibitors [288, 289]. By targeting TOPK kinase, ADA-07 inhibits AP-1 activity by suppressing the phosphorylation of ERK1/2, p38, and JNKs [290]. Indeed, the results of the previous investigation were so supportive that a combined study with RAF inhibitors is currently underway. A recent study showed that inhibiting MAP4K2 with BAY61-3606 can sensitize *KRAS* wild-type colorectal cancer cells to AZ628, a RAF kinase inhibitor, suggesting a potential therapeutic strategy for treating colorectal cancer [291]. Moreover, a noteworthy finding is that the combination of volasertib and LXH254 has shown superiority over LXH254 monotherapy in suppressing long-term cell viability [283]. Furthermore, combination therapy of Raf265 and 5-FU promotes anti-tumor and anti-metastatic activity in colorectal cancer by targeting CD26^+^ tumor stem cells [292]. Regorafenib, a multi-target inhibitor of VEGFR1/2/3, PDGFRβ, and CRAF, has emerged as a notable systemic treatment for HCC patients who had recently received sorafenib treatment, indicating that multi-target inhibition holds tremendous potential in reducing the emergence of resistance [48].

## Conclusions and outlook

Significant progress has been made in the field of CRAF research over the past three decades since its initial discovery. We possess a firm intellectual foundation regarding its involvement in RAS-MAPK signaling and an extensive familiarity with the primary signaling inputs that regulate its function. Although current selective small-molecule CRAF inhibitors possess limitations, they offer hope for controlling unchecked CRAF catalytic/allosteric activity to improve the anti-cancer effect. The rapid accumulation of structural data on members of the RAF family has revealed the intricacies of catalytic switching and may facilitate the discovery of highly specific RAF inhibitors in the future.

 Since its discovery, the intricate regulation of CRAF and its crucial role in human health have captivated researchers. Given the challenges and potential benefits, we expect continued intense exploration in the coming years. Concerning unresolved issues, ongoing efforts in the following areas are expected to yield fruitful results. One area of research focuses on understanding the structure of CRAF proteins, including the role of the N-terminal region and the kinase structural domain in the interaction with RAS-RAF RBD. Furthermore, the exploration of allosteric inhibitors with the capability to disrupt RAF/MEK interactions holds promise as an attractive avenue for future research. In contrast to the first- and second-generation RAF inhibitors, next-generation allosteric inhibitors should have many more advantages, such as circumventing the paradoxical effect and producing fewer off-target effects. Recent findings have underscored the significance of RAF dimerization in various cellular contexts, particularly with its role in resistance linked to inactivation and signal transduction mechanisms. The CRAF DIF mutation (p.R401H), which mirrors the BRAF p.R509H mutation, was found to significantly reduce MEK activation despite facilitating the formation of CRAF homodimers. This finding suggests that RAF-mediated MEK activation relies on a dual-pronged mechanism that involves both dimerization and DIF-dependent transactivation. From a structural standpoint, understanding the RAF dimer interface (DIF) mechanism is crucial. This insight is essential not only in understanding how dimerization affects inhibitor activation but also in guiding the design of future ATP-competitive and allosteric RAF kinase inhibitors. Furthermore, a comprehensive understanding of RAF homo- and hetero-dimerization is crucial. Future studies could guide the design of specialized RAF dimer interface inhibitors that selectively target heterodimers vital for oncogenic signaling while preserving those essential for physiological processes.

Regulation of CRAF by HSP90 and 14-3-3 proteins, and the modulation of downstream apoptotic effectors, such as ROK-α, ASK1, and MST2 by CRAF, are also important areas of investigation. Although CRAF is not crucial for ERK activation, its significance in tumorigenesis and cancer progression largely arises from interactions and cross-talk with other signaling pathways, culminating in non-oncogene addiction in RAS-driven lung cancers and PDAC [74, 85]. Although these alternative inhibitors that target kinase-independent functions may present a safer and more effective therapeutic option, the multi-targeted nature of scaffold proteins introduces a challenge: how to strike a balance between potential multi-targeting toxicities and leveraging their tumor-suppressive attributes. Another pertinent field of research that warrants attention is combining pan-RAF inhibitors with other targeted therapies, such as immune checkpoint inhibitors, EGFR tyrosine kinase inhibitors, or MEK inhibitors. By investigating the aforementioned combination strategies, researchers will likely develop more effective therapeutic approaches for cancer treatment.

The importance of targeting CRAF therapeutically has been substantiated through the validation of new mouse models for *KRAS*/*Trp53*-mutant pancreatic and lung cancers. Moreover, systemic elimination of CRAF in adult mice has shown no substantial toxicities, which contrasts with previous reports of toxicity observed upon ablation of its downstream MEK1/2 and ERK1/2 kinases [81, 80, 85]. However, it is crucial to note that this approach may not universally apply to all *KRAS*-driven cancers. This has been demonstrated with the use of KRAS^G12C^ inhibitors, which have shown limited efficacy in treating colon cancer. Given the well-known challenges of blocking protein-protein interactions with small molecules, pharmacological degradation of CRAF may produce more favorable outcomes. Recent research in targeted protein degradation may present opportunities for the selective degradation of CRAF. Promising prospects include using molecular chaperone-mediated protein degraders (CHAMP) and proteolysis-targeting chimera (PROTAC). Identifying compounds that can bind to domains exclusive to CRAF isoforms makes it possible to target and specifically degrade CRAF [293]. In sum, although the inhibition of CRAF has exhibited promising results in preclinical studies, the clinical development of CRAF inhibitors is still in its early stages. However, with ongoing progress in innovative technologies and increasing comprehension of the intricacies of KRAS signaling, there is growing optimism that targeting CRAF may constitute a pivotal component of cancer therapies.

## Data Availability

Not applicable.

## Abbreviations

BLCA: Bladder Urothelial Carcinoma
SKCM: Skin Cutaneous Melanoma
DLBC: Lymphoid Neoplasm Diffuse Large B-cell Lymphoma
UCEC: Uterine Corpus Endometrial Carcinoma
STAD: Stomach adenocarcinoma
ESAD: Esophageal adenocarcinoma
SARC: Sarcoma
KIRC: Kidney renal clear cell carcinoma
COAD: Colon adenocarcinoma
THCA: Thyroid carcinoma
BRCA: Breast invasive carcinoma
LUAD: Lung adenocarcinoma
LIHC: Liver hepatocellular carcinoma
KIRP: Kidney renal papillary cell carcinoma
OV: Ovarian serous cystadenocarcinoma
CESC: Cervical squamous cell carcinoma and endocervical adenocarcinoma
LGG: Brain Lower Grade Glioma
LUSC: Lung squamous cell carcinoma
THYM: Thymoma
PRAD: Prostate adenocarcinoma
GBM: Glioblastoma multiforme
TGCT: Testicular Germ Cell Tumor
HNSC: Head and Neck squamous cell carcinoma
PCPG: Pheochromocytoma and Paraganglioma
GFR: Growth Factor Receptor
bFGF: basic Fibroblast Growth Factor
DC-SIGN: Dendritic Cell-Specific Intercellular adhesion molecule-3-Grabbing Non-integrin
TLR: Toll-like Receptor
TRIF: TIR-domain-containing Adapter-inducing Interferon-β
NIK: NF-κB-Inducing Kinase
CAV1: Caveolin-1
HSP90: Heat Shock Protein 90
ROK-α: Rho-Associated Coiled-Coil Kinase Alpha
AURKA: Aurora Kinase A
PRMT6: Protein Arginine Methyltransferase 6
PKM2: Pyruvate Kinase M2
PHLPP1/2: PH Domain and Leucine-Rich Repeat Protein Phosphatases 1/2
PKA: Protein Kinase A
MAZ: MYC-Associated Zinc Finger Protein
PLK-1: Polo-Like Kinase 1
AKT: Protein Kinase B
TGF-β: Transforming Growth Factor-beta
EGFR: Epidermal Growth Factor Receptor
PI3K: Phosphoinositide 3-Kinase
STAT: Signal Transducer and Activator of Transcription
mTOR: Mammalian Target of Rapamycin
PD-1/PD-L1: Programmed Cell Death Protein 1/Programmed Death-Ligand 1
CTLA-4: Cytotoxic T-Lymphocyte-Associated Protein 4
